# Ripening and thermal processing modulate starch digestibility, techno-functional properties and carotenoid profile in cooking banana cultivars (*Musa* spp.)

**DOI:** 10.1016/j.fochx.2026.104678

**Published:** 2026-10-27

**Authors:** Jhon Larry Moreno-Alzate, Santiago Arufe, Thierry Tran, Moralba Dominguez, Dominique Dufour

**Affiliations:** a The Alliance of Bioversity International and the International Center for Tropical Agriculture (CIAT), Apartado Aereo, Cali 6713, Colombia; b CIRAD, UMR Qualisud, F-34398 Montpellier, France; c Qualisud, Avignon Université, CIRAD, Institut Agro, IRD, Université de Montpellier, Université La Réunion, Montpellier, France

**Keywords:** Food functionality, Nutritional value, Low glycemic food, HPLC, RVA, Thermal processing, Cooking banana flour

## Abstract

This study evaluated the combined effects of ripening stage (unripe, ripe) and boiling on carotenoids, starch digestibility, estimated glycemic index (eGI), pasting properties, and sugars across four Colombian cooking banana cultivars.

Dominico Hartón exhibited the highest provitamin A carotenoid content (1667 μg/100 g f.w.), dominated by trans- β-carotene. Unripe raw flour showed high resistant starch (up to 71%) and low eGI (<60). Three bunches per genotype were harvested as biological replicates for carotenoid and dry matter analyses; starch and functional analyses were performed in analytical duplicates Conversely, ripening and boiling substantially increased rapidly digestible starch and eGI (>80) due to starch gelatinization.

Genotype, ripening, and processing jointly dictate the nutritional value and functional performance of cooking banana flours. Selecting suitable cultivars allows the development of low-glycemic functional ingredients, contributing to food security and public health in tropical regions facing vitamin A deficiency.

## Highlights

• Unripe cooking banana flours contain high resistant starch (up to 71%).
• Boiling and ripening melt starch structure, increasing RDS and eGI (>80).
• Dominico Hartón shows high provitamin A content (1667 μg/100 g f.w.).
• 3-way interactions (cultivar × ripening × boiling) dictate paste properties.
• Enables tailored selection of low-glycemic functional ingredients.

## Graphical abstract

**Figure:**
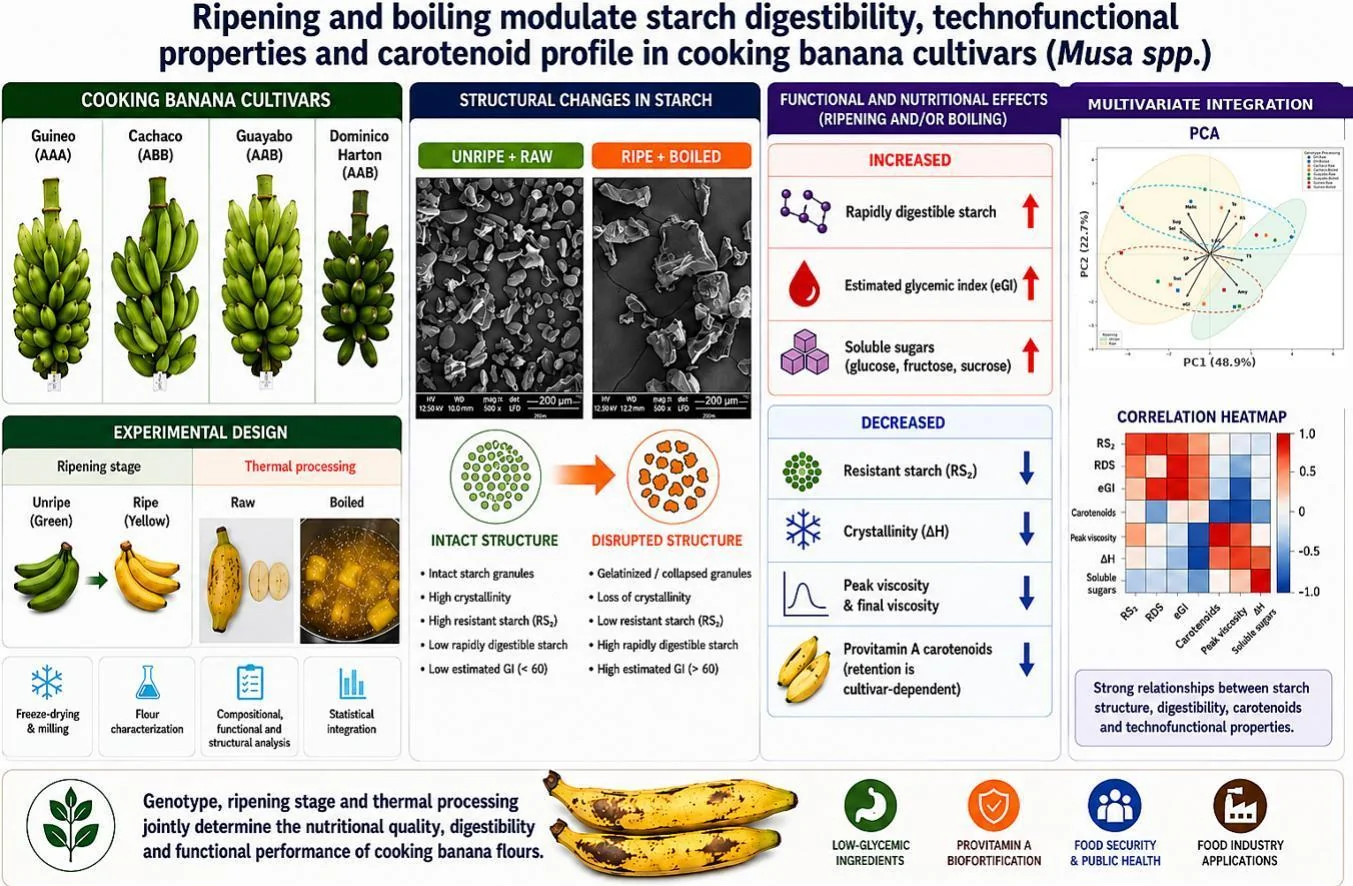

## 1 Introduction

Cooking bananas and plantains (*Musa* spp.) represent vital staple crops that underpin food security and income for millions of smallholder farmers in tropical regions, with annual production exceeding 42 million tonnes (Lescot, 2020). Colombia is the leading producer in the Americas, harboring extensive genetic diversity across locally cultivated genotypes (Lescot, 2020). Unlike dessert bananas, which rapidly convert starch into soluble sugars during postharvest ripening, cooking bananas retain high starch reserves even at advanced maturity, needing thermal processing before consumption (Dadzie & Orchard, 1997; Moreno et al., 2021). This physiological divergence imparts higher dry matter, firmer cooked texture, and distinct processing attributes (Moreno et al., 2021). Although starch content influences the physical properties of the tissue, cell wall stability is the main factor responsible for resistance to softening. Modifications to pectins, particularly the decrease in the molecular size of the polymers and the loss of cohesion in the middle lamella, explain much of the rapid disintegration observed in bananas compared to plantains (Qi et al., 2000).

Starch functionality and nutritional value are governed by molecular organization (amylose-to-amylopectin ratio) and hierarchical granule architecture, which dictate gelatinization, swelling power, retrogradation, and enzymatic susceptibility (Dhital et al., 2016; Hoover, 2001; Wang & Copeland, 2013). Unripe cooking bananas are particularly rich in resistant starch (RS2), which escapes small-intestinal digestion and contributes to glycemic regulation and gut health through colonic fermentation (Campuzano et al., 2018; Chang et al., 2022). Germplasm characterizations across international collections (Amah et al., 2019; Davey et al., 2007; Drapal et al., 2024; Ngoh Newilah et al., 2021; Udomkun et al., 2020) and native Colombian accessions (Dufour et al., 2009; Enriquez-Valencia et al., 2023; Hoyos-Leyva et al., 2012) have revealed substantial genetic variability in starch thermal profiles (DSC), pasting behavior (RVA), microstructure (Jane et al., 1999; Qi et al., 2000), and water interactions. Beyond carbohydrate metabolism, cooking bananas are important sources of provitamin A carotenoids, whose bioaccessibility and Retinol Activity Equivalents (RAE) are strongly affected by matrix disruption and thermal processing (Englberger et al., 2010; Rodriguez-Amaya et al., 2006). Simultaneously, consumer acceptability and culinary quality are heavily governed by the dynamic equilibrium between soluble sugars and organic acids primarily malic, citric, oxalic, and succinic acids (Etienne et al., 2014; Gibert et al., 2013; Vermeir et al., 2009). Organic acids directly modulate pulp pH and titratable acidity, acting as key drivers of overall taste perception, sweetness-to-tartness balance, and flavor acceptability (Etienne et al., 2014). Shifts in organic acid concentrations during postharvest ripening and their subsequent modifications during cooking (via thermal degradation, leaching, and enzyme inactivation) fundamentally dictate the final organoleptic profile, directly shaping consumer preferences and traditional food choices (Etienne et al., 2014; Vermeir et al., 2009). Beyond these organic constituents, potassium is also highly abundant in *Musa* spp. fruit, varying widely by genotype and ripening stage (Borges et al., 2020).

However, previous research on Colombian cultivars has focused primarily on starch properties or cooking quality in isolation, mostly restricted to mature-green fruit (Gibert et al., 2010, Giraldo Toro et al., 2016). To date, no study has integrated starch microarchitecture, in vitro digestibility, estimated glycemic index, carotenoid retention, and organic acid profiles within a single set of cultivars evaluated across ripening stages under both raw and cooked conditions (Gibert et al., 2010, Giraldo Toro et al., 2016).

To address this gap, this study evaluated the combined effects of ripening stage (unripe vs. ripe) and thermal processing (boiling) on four representative Colombian cooking banana cultivars (Dominico Harton, Cachaco, Guayabo, and Guineo). The specific objectives were to: (i) quantify carotenoid composition and RAE in raw and boiled pulp; (ii) characterize starch structure, thermal and pasting properties, microstructure, in vitro digestibility, estimated glycemic index, and soluble sugar/organic acid profiles in the corresponding flours; and (iii) elucidate the interrelationships among the evaluated parameters of nutritional and techno-functional relevance.

## 2 Materials and methods

### 2.1 Plant material

Four cooking banana genotypes were used in this study, all harvested in San Isidro, Palmira, Valle del Cauca, Colombia (latitude 03°28′22.9“ N, longitude 76°19’20.7” W, altitude 994 m.a.s.l.): Dominico Harton (AAB, Plantain subgroup), Cachaco (ABB, Bluggoe subgroup), Guayabo (AAB, Maoli-Popouluf/Maia Maoli subgroup), and Guineo (AAA-EAHB, Mutika-lujugira subgroup), all classified as cooking type. Three bunches were harvested per genotype and fruits from hands 2 and 3 were used. The fruits were divided into two ripening stages during post-harvest storage according to the banana color scale described by Soltani et al. (2011), based on peel color: green (unripe) and completely yellow (ripe). Once harvested, raw and boiled fruits were at once processed and analyzed, while the remaining fruits were stored under ambient conditions (25–28 °C, 56–62% relative humidity) until fully yellow.

### 2.2 Fruit processing

Fruits from hands 2 and 3 were pooled per bunch and randomly divided for fresh and boiling treatments. Fresh fruits were peeled, grated and analyzed at once. For boiling, peeled fruits were cut into cylinders (approx. 3.5 cm diameter × 4 cm length). This sampling method was used to assure the same dimensions of the samples to reduce the number of factors that may cause variability in the results. Six cylinders per cultivar were boiled in 700 mL deionized water in a 1000 mL beaker for 20 min at boiling temperature (duplicate). Cooked material was cooled for 20 min, then grated and homogenized. Subsamples were taken for dry matter and carotenoid analyses. All samples and extracts were protected from light to avoid photo degradation of the carotenoid pigments.

### 2.3 Flour production

Raw and boiled pulps were freeze-dried, ground through a 1 mm sieve using a Foss mill (CT 293 Cyclotec™) and stored at −80 °C until analysis.

### 2.4 Characterization of raw and boiled pulp

#### 2.4.1 Dry matter content

A pulp sample (either raw or boiled) was taken to quantify the dry matter content. 3 g of the pulp was weighed, this was done in duplicate for each cluster, and it was dried in an oven (Blue M, OV-510 A-2, USA) at 105 °C for 24 h. Dry matter was expressed as the percentage of dry weight in relation to fresh weight.

#### 2.4.2 Extraction of carotenoids

Carotenoids were extracted according to Ceballos et al. (2012) with minor modifications. Pulp samples (5 g, raw or boiled) were placed in falcon tubes with 10 mL acetone and allowed to stand for 10 min. Petroleum ether (10 mL) was then added, and the mixture was homogenized with an Ultra-Turrax (IKA, T25 DS1, Germany) for 30 s, followed by centrifugation (Eppendorf, 5804R, Germany) at 7000 rpm for 7 min at 10 °C. The organic phase was collected, and the extraction was repeated twice on the remaining sediment with 5 mL acetone and 5 mL petroleum ether until the residues were colorless. The pooled organic phases were washed twice with 10 mL of 0.1 M NaCl and centrifuged at 7000 rpm for 5 min at 10 °C. The aqueous phase was discarded, and the final extract volume was adjusted to 20 mL with petroleum ether. All operations were performed under dim light, with samples wrapped in aluminum foil and laboratory lights fitted with UV filters to prevent carotenoid photodegradation.

#### 2.4.3 Analysis of carotenoids by reverse phase high-performance liquid chromatography (HPLC) with diode array detector (DAD)

For HPLC-DAD analysis, the organic extract was evaporated to dryness under nitrogen (Organomation Associates, N-Evap 112, USA), reconstituted in 2 mL of methanol: methyl tert-butyl ether (1:1, *v*/v), vortexed for 60 s at 2400 rpm, and filtered through a 0.22 μm PTFE membrane filter prior to injection. Carotenoid separation was performed on a YMC Carotenoid S-5 C30 reverse-phase column (4.6 × 150 mm, 5 μm particle size) equipped with a YMC Carotenoid S-5 guard column (4.0 × 23 mm), using an Agilent Technologies 1200 series HPLC system (Germany) with DAD detection at 450 nm. Carotenoids were identified by comparing retention times and UV–visible spectral characteristics (λ_max_, fine structure, and peak intensity) against certified reference standards from CaroteNature GmbH (Lupsingen, Switzerland): lutein (No. 0133, ≥97%), α-carotene (No. 0007, ≥97%), and all-trans β-carotene (No. 0003, ≥96%). The 9-cis, 13-cis, and 15-cis β-carotene isomers were tentatively identified based on elution order and spectral features and quantified using the all-trans β-carotene calibration curve. Individual carotenoid contents were determined by external standard calibration, and total carotenoids were estimated from the sum of all peak areas using the all-trans β-carotene response factor.

Retinol activity equivalent (RAE) was calculated as:

(1) $$\mathrm{RAE}\;\left(\mathrm{\mu g}\right)=\left[t-\mathrm{\beta C}/12\right]+\left[\mathrm{\alpha C}/24\right]+\left[9\;\mathrm{cis}-\mathrm{\beta C}/24\right]+\left[13\;\mathrm{cis}-\mathrm{\beta C}/24\right]+\left[15\;\mathrm{cis}-\mathrm{\beta C}/24\right]$$

Where t-βC = all-trans-β-carotene, αC = α-carotene and cis-βC = cis isomers of β-carotene (Yeum & Russell, 2002).

### 2.5 Flour characterization

The dry matter content of the flours was determined following the methodologies described in Section 2.4.1.

#### 2.5.1 Amylose determination and gelatinization properties

The methodology described by Mestres et al. (1996) was applied. Differential scanning calorimetry (DSC) analyses were performed using a Perkin-Elmer DSC 7 instrument (Perkin Elmer, USA) and hermetically sealed stainless steel pans. For the determination of amylose content, approximately 10–11 mg of flour sample was used, to which 50 μL of 2% (*w*/*v*) lysophospholipid in water was added to the sample pan, while the reference pan remained empty. Both pans were subjected to a thermal program consisting of heating from 25 to 160 °C at a rate of 10 °C min^−1^, followed by holding at 160 °C for 2 min and later cooling to 60 °C at 10 °C min^−1^. Amylose content was estimated from enthalpy associated with the formation of the amylose-lysophospholipid complex. For the determination of thermal properties, a similar amount of sample was used, replacing the lysophospholipid with water. The thermal program included an initial equilibration at 25 °C for 2 min, followed by heating from 25 to 140 °C at a rate of 10 °C min^−1^, and finally, cooling from 140 to 25 °C at a rate of 50 °C min^−1^.

#### 2.5.2 Total starch content

Total starch content was determined according to the method of Holm et al. (1986) with some modifications. Firstly, total carbohydrates were determined after incubation with thermostable α-amylase (L AYN04010 type, Novo Nordisk, Denmark) and then with amyloglucosidase (70 U/mg, Sigma Ref.10115). The released glucose was measured with a spectrophotometer at 510 nm, after reaction with GOPOD containing glucose oxidase (Sigma, G6125-50KU) and peroxidase (Sigma; P8112-25KU). Secondly, free glucose was determined after incubation with amyloglucosidase and again released glucose is measured on a spectrophotometer (510 nm) after reaction with GOPOD solution. Total starch content was calculated using the following equation:

(2) $$\mathrm{TS}=C\;x\;\left(162/180\right)–\mathrm{FG}$$

where TS is total starch (% *w*/w, db); C is total carbohydrates (% *w*/w, dry basis); 162/180 is the conversion factor from free glucose, as experimentally determined, to anhydro glucose units present in starch; and FG is free glucose (% *w*/w, dry basis).

#### 2.5.3 In-vitro kinetic study of starch hydrolysis

The in vitro digestibility of starches was determined by the method of Englyst et al. (1992), with some modifications. Starch samples (0.6 g) and guar gum (0.06 g) with Milli-Q water (6 mL) were added to 50 mL polypropylene centrifuge tubes, followed by addition of 12 mL pepsin solution (Sigma, P7000) in 0.01 M HCL (5 mg / mL). The tubes were incubated at 37 °C for 30 min with continuous shaking (160 strokes / min). Five glass beads (diameter 3–4 mm) and 2.5 mL sodium acetate buffer (0.5 M, pH 6) were added, and the tubes were vortexed for 20 s. The dispersion was then mixed with an enzyme solution (2.5 mL) consisting of pancreatin extract (Sigma, P-7545), amyloglucosidase (Sigma, A7095, 300 U/mL), and invertase (Sigma, I4504, 300 U/mL), the tubes were again incubated at 37 °C. The hydrolyzed glucose content was measured using the GOPOD solution at different times (0, 20, 50, 80 and 120 min). A volume of 100 μL of the dispersion was collected and immediately vortexed with 1.5 mL of ethanol 75% (*v*/v) to stop the hydrolysis.

The digested starch fractions were expressed in % of glucose x 0.9. Rapidly digestible starch (RDS) and slowly digestible starch (SDS) were calculated from the values measured at 20 and 120 min of hydrolysis. The resistant starch (RS) was calculated using the following equation:

(3) RS:TS−RDS−SDS

where TS is the total starch content.

The in vitro digestibility curve of the samples can be fitted to a first-order equation (Butterworth et al., 2012):

(4) $$\mathrm{Ct}=\mathrm{C\alpha}\;\left(1-e^{-\mathrm{kt}}\right)$$

Simultaneously, the logarithmic slope (LOS) plot (the relationship between ln(1 − Ct/C∞) on the vertical Y-axis and t on the horizontal X-axis) was used to analyze starch hydrolysis data.

Where Ct is the fraction of starch digested at time t (mim), Cα is the extent of starch hydrolysis at the end of the reaction; *K* is the first-order rate constant for in vitro starch hydrolysis (min^−1^). The value of K was obtained from the slope of the linear least squares fit of LN(1 − *C*) versus *t* (Zhang et al., 2013). The area under the hydrolysis curve (AUC) was calculated by integrating the kinetic equation. The hydrolysis index (HI) was then determined as the ratio between the AUC of the starch samples and that of a reference sample (white bread). Finally, the estimated glycemic index (eGI) was calculated using the equation proposed by Goñi et al. (1997).

(5) eGI=39.71+0.549HI

#### 2.5.4 Pasting properties

Pasting properties were determined using a Rapid Visco Analyzer (Perten Instruments, RVA 4500; Australia). 2.0 g of flour (dry basis) was weighed, and a 0.002 M silver nitrate solution was added to a total weight of 25 g, resulting in an 8% (*w*/w) suspension, according to the method described by Dufour et al. (2009). Viscosity was continuously recorded using the following temperature profile: equilibration at 50 °C for 1 min, heating from 50 to 90 °C at a rate of 6 °C min^−1^, holding at 90 °C for 5 min, cooling from 90 to 50 °C at 6 °C min^−1^, and a final holding period at 50 °C for 2 min. Throughout the test, the stirring speed was kept constant at 160 rpm. Four parameters were measured in the viscoamylogram: pasting temperature (PT), peak viscosity (PV), Trough (Tr), and final viscosity (FV). Two additional parameters were then calculated: breakdown (BD) estimated as PV-Tr and setback (SB) estimated as FV-Tr.

#### 2.5.5 Solubility and swelling power

Solubility and swelling power were determined by the method reported by Aparicio-Saguilán et al. (2005), with some modifications. Starch dispersions were used at 1% (*w*/w) on a dry basis (0.28 g dispersed in 27.72 g of silver nitrate solution at 0.002 M). The samples were transferred to a boiling water bath for 30 min. The samples were then cooled to room temperature and centrifuged for 25 min at 7000 rpm and 25 °C. The supernatant and sediment were separated and weighed (W_so_ and W_se_, respectively), then dried at 105 °C for 48 h and reweighed (D_so_ and D_se_, respectively). The values obtained were used to calculate two parameters: concentration of soluble material in the supernatant (solubility) and swelling power, using the following equations:

(6) $$\text{Solubility}\;\left(\%\right)=100*D_{\mathrm{so}}\;0.28$$

(7) $$\text{Swelling power}\;\left(g_{\text{water}}/g_{\text{starch}}\right)=.\left(W_{\mathrm{se}}-D_{\mathrm{se}}\right)/D_{\mathrm{se}}$$

#### 2.5.6 Organic acids and sugars

The sample (0.5 g) was weighed and 10 mL of 5 mM H₂SO₄ solution was added. The mixture was homogenized using an Ultra-Turrax homogenizer (IKA, T25 DS1, Germany) for 30 s and then shaken in a multi-tube vortex mixer (Fisher brand, 945091, USA) at 2400 rpm for 1 h. The suspension was then centrifuged (Eppendorf, 5804 R, Germany) at 8000 rpm for 10 min at 25 °C. The supernatant was filtered through a 0.22 μm PVDF membrane filter (Millex-GV, Brazil), transferred to 1.5 mL vials, and placed in the instrument's autosampler for analysis. Organic acids and sugars were quantified by high-performance liquid chromatography (HPLC) using a Bio-Rad Aminex HPX-87H column equipped with a UV detector (Agilent Technologies, MWD T1365D, Germany) set to 210 nm for the determination of organic acids (oxalic, citric, malic, and succinic) and connected in series with a refractive index detector (Agilent Technologies, RID T1362A, Germany) for the analysis of sugars (glucose, fructose, sucrose and raffinose). The equipment features a quaternary injection valve. Samples were separated isocratically at a flow rate of 0.6 mL/min, 30 °C, and an injection volume of 15 μL. All reagents used in the HPLC analysis were of analytical grade. Concentrations were calculated using calibration curves that related the concentration of each compound to the corresponding peak area (Moreno, 2011).

#### 2.5.7 Light microscopy and polarizing microscopy

For the morphological characterization of starch granules, an Optika B-510POL-I optical microscope (Ponteranica, Italy) was used, equipped with an Optika B10 digital camera, which allowed for the capture of high-resolution images. The images were obtained using Optika ProView software (64-bit version, 4.10.16880.2020030), which facilitated their acquisition, storage, and basic processing. The samples were prepared by placing a small amount of flour on a glass slide; later, two drops of deionized water were added. The mixture was gently homogenized and covered with coverslip, taking care to avoid the formation of bubbles and ensure adequate microscopic visualization. Observations were made under bright-field and polarized white light illumination, using a 20× objective, selected for providing optimal magnification that allowed for clear distinction of the contours and structural characteristics of the granules.

#### 2.5.8 Scanning electron microscopy

Micrographs of native starches were obtained by scanning electron microscopy as described by Yan and Zhengbiao (2010). Scanning electron micrographs of native and modified starches were obtained with a scanning electron microscope (Quanta-200, FEI Ltd., Holland). Starch samples were sprinkled on double stick tape fixed on an aluminum stub, and the starch was coated with gold–palladium (60:40).

### 2.6 Statistical analysis

Statistical analyses were performed in order to determine the effect of three fixed factors: cultivar (C; Dominico Hartón, Cachaco, Guayabo, and Guineo), maturity stage (R; unripe and ripe), and processing (P; raw and boiled). The model included the three-way interaction C x R x P. The assumptions of normality of residuals and homogeneity of variances were assessed using the Shapiro–Wilk and Levene tests, respectively (α = 0.05) in order to determine the ANOVA model to be applied to the data.

When both assumptions were verified (Dry matter), the effects of C, R, and P and their interactions were evaluated using a three-way factorial ANOVA (Type III sums of squares), and treatment combination means were compared using Tukey's HSD test. When normality was not met (Carotenoids) or for variables with *n* = 2 (two replicates per treatment), implying that Levene's test cannot be applied, rank-based procedures were applied to all variables: effects were evaluated using ART, and means were compared using the Tukey–Kramer test on rank-transformed (RT) data.

For dry matter and carotenoids (Table 1), *n* = 3 corresponds to three independent biological replicates (three distinct bunches per cultivar). For starch digestibility, organic acid and sugar content, and pasting, hydration, and thermal properties (Table 2, Table 3, Table 4), *n* = 2 corresponds to two independent processing replicates obtained from a single composite pulp sample for each cultivar–maturity stage combination; therefore, these are analytical rather than biological replicates. In the ART procedure, the response was aligned for each effect and transformed into ranks; the full factorial model was then fitted, and only the corresponding aligned effect was interpreted.

**Table 1:** Dry matter, lutein and provitamin A carotenoids (t-βC, αC, 15-c-βC, 13-c-βC and 9-c-βC) content, total carotenoids, total pVACs (provitamin A carotenoids) and retinol activity equivalent (RAE) in raw and boiled cooking bananas at different ripening stages.

Local name of Cultivar	Ripening stage	Processing	Dry matter (%, fw)	pVACs (μg/ 100 g fw)					Lutein (μg/ 100 g fw)	Total Carotenoids (μg/ 100 g fw)	pVACs (μg/ 100 g fw)	RAE (μg/ 100 g fw)
				**t-βC**	**αC**	**15-c-βC**	**13-c-βC**	**9- c-βC**				
Dominico Harton	Unripe	Raw	41.2 ± 0.2^a^	926.0 ± 12.6^a^	502.2 ± 12.3^a^	4.5 ± 0.1^fg^	14.5 ± 0.6^ef^	1.5 ± 0.1^ef^	7.7 ± 0.9^gh^	1666.6 ± 28.3^a^	1448.7 ± 24.4^a^	98.9 ± 1.5^a^
	Unripe	Boiled	38.3 ± 0.7^bc^	343.5 ± 97.9^cde^	186.6 ± 48.4^abc^	16.9 ± 4.6^abc^	60.4 ± 19.0^ab^	3.7 ± 1.2^de^	16.2 ± 1.4^efg^	771.9 ± 196.8^bcd^	611.1 ± 170.7^abc^	39.8 ± 11.2^abcd^
	Ripe	Raw	38.2 ± 0.2^bc^	339.7 ± 20.4^bc^	194.0 ± 8.4^abc^	6.5 ± 1.7^f^	21.8 ± 5.4^de^	10.1 ± 3.2^cd^	16.4 ± 1.3^efg^	723.6 ± 39.5^abc^	572.0 ± 32.6^abc^	38.0 ± 2.2^abc^
	Ripe	Boiled	33.2 ± 0.8^d^	250.9 ± 7.8^efgh^	149.2 ± 6.8^bcd^	12.5 ± 1.2^bcd^	49.8 ± 5.9^bc^	36.3 ± 14.2^a^	33.8 ± 2.8^bc^	701.7 ± 47.6^abc^	498.7 ± 34.5^bcd^	31.2 ± 1.8^de^
Cachaco	Unripe	Raw	39.3 ± 0.4^ab^	531.3 ± 20.5^ab^	73.8 ± 1.0^efg^	1.8 ± 0.4^g^	ND^g^	1.0 ± 0.7^efg^	4.5 ± 0.7^h^	704.4 ± 22.0^abc^	607.8 ± 20.9^ab^	47.5 ± 1.7^ab^
	Unripe	Boiled	36.9 ± 0.5^c^	319.6 ± 10.8^bcd^	50.0 ± 0.3^hi^	18.2 ± 1.1^ab^	70.0 ± 2.7^ab^	11.0 ± 2.7^bcd^	18.6 ± 0.8^de^	563.7 ± 12.5^defg^	468.7 ± 12.4^de^	32.8 ± 1.0^cde^
	Ripe	Raw	37.5 ± 0.5^bc^	480.3 ± 46.1^abc^	75.3 ± 6.2^efg^	8.4 ± 1.1^def^	46.4 ± 9.7^bc^	10.7 ± 3.4^bcd^	18.2 ± 3.0^def^	741.5 ± 58.4^ab^	621.0 ± 38.0^a^	45.9 ± 3.5^ab^
	Ripe	Boiled	30.0 ± 1.4^ef^	310.1 ± 13.1^cde^	51.4 ± 0.8^ghi^	23.1 ± 2.0^a^	79.1 ± 6.7^a^	25.9 ± 3.2^ab^	14.9 ± 0.2^fgh^	607.1 ± 28.8^cdef^	489.7 ± 25.0^cde^	33.3 ± 1.6^bcd^
Guayabo	Unripe	Raw	33.2 ± 1.2^d^	268.4 ± 31.7^defg^	110.2 ± 10.3^de^	ND^h^	ND^g^	0.6 ± 0.7^fg^	6.8 ± 0.4^h^	467.1 ± 53.2^efgh^	379.2 ± 42.3^ef^	27.0 ± 3.1^ef^
	Unripe	Boiled	27.8 ± 0.3^g^	150.3 ± 24.0^hijk^	66.6 ± 6.2^fgh^	8.0 ± 1.8^ef^	23.1 ± 7.1^de^	3.1 ± 1.9^ef^	25.7 ± 1.6^cd^	362.9 ± 44.4^ghi^	251.2 ± 40.7^fg^	16.7 ± 2.7^fg^
	Ripe	Raw	31.1 ± 0.3^e^	175.9 ± 67.1^ghij^	65.4 ± 18.4^gh^	1.2 ± 1.1^gh^	7.9 ± 7.5^fg^	4.3 ± 2.6^de^	28.3 ± 10.1^cd^	400.9 ± 61.1^fghi^	254.8 ± 96.5^fg^	17.9 ± 6.8^fg^
	Ripe	Boiled	28.0 ± 0.8^fg^	269.4 ± 26.4^def^	90.1 ± 8.3^def^	18.2 ± 2.5^a^	60.8 ± 9.2^ab^	25.4 ± 3.1^abc^	38.9 ± 3.4^abc^	624.5 ± 58.6^cde^	464.0 ± 48.3^de^	30.6 ± 3.1^de^
Guineo	Unripe	Raw	30.0 ± 0.1^ef^	26.7 ± 1.4^k^	42.5 ± 3.1^i^	ND^h^	ND^g^	ND^g^	25.9 ± 3.2^cd^	109.9 ± 8.8^i^	69.2 ± 4.3^g^	4.0 ± 0.2^g^
	Unripe	Boiled	24.8 ± 0.8^h^	32.4 ± 7.2^jk^	43.2 ± 5.9^i^	ND^h^	ND^g^	ND^g^	64.6 ± 11.5^ab^	174.5 ± 33.6^hi^	75.6 ± 13.2^g^	4.5 ± 0.8^g^
	Ripe	Raw	21.8 ± 0.3^i^	126.6 ± 9.5^ijk^	136.5 ± 9.7^cd^	ND^h^	ND^g^	ND^g^	64.9 ± 3.5^ab^	393.0 ± 24.7^fghi^	263.1 ± 18.5^fg^	16.2 ± 1.2^fg^
	Ripe	Boiled	16.7 ± 0.2^j^	182.8 ± 15.6^fghi^	222.3 ± 9.3^ab^	12.1 ± 1.4^cde^	34.7 ± 5.6^cd^	12.8 ± 1.4^abc^	88.6 ± 5.5^a^	750.2 ± 45.5^ab^	464.7 ± 22.4^de^	27.0 ± 1.6^ef^


**Statistical significance of the sources of variation III SS**										
Local name of cultivar (C)	< 0.001 ***	< 0.001 ***	< 0.001 ***	< 0.001 ***	< 0.001 ***	< 0.001 ***	< 0.001 ***	<0.001 ***	<0.001 ***	< 0.001 ***
Ripening stage (R)	< 0.001 ***	< 0.001 ***	< 0.001 ***	< 0.001 ***	< 0.001 ***	< 0.001 ***	< 0.001 ***	0.2088 ns	0.0096 **	< 0.001 ***
Processing (P)	< 0.001 ***	< 0.001 ***	< 0.001 ***	< 0.001 ***	< 0.001 ***	< 0.001 ***	< 0.001 ***	<0.001 ***	<0.001 ***	< 0.001 ***
Interaction (CxRxP)	< 0.001 ***	< 0.001 ***	< 0.001 ***	< 0.001 ***	< 0.001 ***	< 0.001 ***	< 0.001 ***	<0.001 ***	<0.001 ***	< 0.001 ***

Values are presented as means ± SD (*n* = 3 biological replicates). Within each column, means followed by different superscript letters differ significantly (α = 0.05). Dry matter tested by parametric three-way ANOVA (Type III SS), and means of the treatment combinations were compared by Tukey's HSD test. Carotenoid variables tested by aligned rank transform ANOVA (ART) and means of the 16 treatment combinations were compared by the Tukey–Kramer test on rank-transformed data (rank transform, RT). ND not detected (treated as zero in the statistical analyses). Significance codes: *** *p* < 0.001; ** *p* < 0.01; * *p* < 0.05; ns, not significant. t-βC, all-trans-β-carotene; αC, α-carotene; 15-c-βC, 15-cis-β-carotene; 13-c-βC, 13-cis-β-carotene; 9-c-βC, 9-cis-β-carotene; pVACs, provitamin A carotenoids; RAE, retinol activity equivalents.

**Table 2:** Amylose content, total starch, in vitro digestion properties, kinetic parameters and estimated glycemic index of flours from different cultivars and processing methods.

Local name of Cultivar	Ripening stage	Processing	Total starch (%, db)	Starch fractions (%, db)			Amylose (%, db)	C_80_ (%, db)	k (min^−1^)	eGI	RMSE (%, db)	R^2^
				**RDS**	**SDS**	**RS**						
Dominico Harton	Unripe	Raw	88.8 ± 1.1^a^	5.1 ± 0.3^ij^	12.6 ± 0.0^def^	71.1 ± 0.2^a^	18.7 ± 0.0^ab^	16.5 ± 0.3^klm^	0.023 ± 0.001^bcdef^	49.1 ± 0.2^jkl^	0.6	0.994
	Unripe	Boiled	70.0 ± 1.9^bcd^	41.4 ± 0.5^ab^	26.5 ± 0.8^ab^	2.1 ± 1.3^ij^	18.7 ± 0.5^ab^	78.7 ± 0.4^def^	0.022 ± 0.001^bcdef^	84.4 ± 0.2^de^	7.6	0.947
	Ripe	Raw	43.4 ± 4.5^ghi^	4.3 ± 0.9^jk^	9.9 ± 0.3^fgh^	29.2 ± 0.6^ef^	6.7 ± 0.4^hij^	32.3 ± 0.7^ghi^	0.067 ± 0.033^ab^	59.7 ± 0.8^fgh^	4.2	0.895
	Ripe	Boiled	33.9 ± 0.9^ij^	31.7 ± 0.3^bcd^	1.7 ± 0.3^gh^	0.5 ± 0.0^jk^	11.6 ± 0.2^efg^	97.3 ± 0.1^bc^	0.053 ± 0.000^abcd^	98.9 ± 0.0^bc^	1.9	0.999
Cachaco	Unripe	Raw	69.9 ± 3.9^bcd^	5.8 ± 0.3^hi^	11.8 ± 0.8^efg^	52.3 ± 1.1^bc^	16.6 ± 0.0^abc^	24.7 ± 0.7^ijk^	0.052 ± 0.020^abcd^	54.9 ± 0.6^hij^	2.4	0.939
	Unripe	Boiled	61.0 ± 4.5^efg^	48.0 ± 0.8^a^	10.5 ± 1.9^fgh^	2.4 ± 1.1^ij^	15.0 ± 0.5^cde^	94.2 ± 0.9^cd^	0.067 ± 0.039^ab^	98.0 ± 4.8^bc^	1.8	0.997
	Ripe	Raw	58.6 ± 2.6^fgh^	11.8 ± 0.3^gh^	13.3 ± 0.0^bcde^	33.5 ± 0.3^de^	8.9 ± 0.3^ghi^	31.5 ± 0.5^hij^	0.018 ± 0.001^def^	57.7 ± 0.2^ghi^	4.3	0.926
	Ripe	Boiled	41.0 ± 4.3^hi^	39.8 ± 0.6^abc^	0.8 ± 0.6^h^	0.4 ± 0.0^lm^	9.9 ± 0.2^fgh^	97.2 ± 1.3^abc^	0.049 ± 0.008^abcd^	98.2 ± 2.1^bc^	0.7	1.000
Guayabo	Unripe	Raw	79.0 ± 0.1^ab^	2.8 ± 0.1^kl^	12.9 ± 0.3^cdef^	63.3 ± 0.1^ab^	15.5 ± 0.0^bcd^	15.1 ± 0.5^lm^	0.019 ± 0.001^cdef^	48.3 ± 0.2^kl^	0.4	0.996
	Unripe	Boiled	77.4 ± 0.9^abc^	22.9 ± 0.0^def^	40.4 ± 1.4^a^	14.1 ± 1.4^gh^	19.1 ± 0.8^a^	81.5 ± 0.0^de^	0.055 ± 0.011^abc^	89.3 ± 1.1^cd^	4.1	0.983
	Ripe	Raw	55.0 ± 4.2^fgh^	0.9 ± 0.3^l^	16.4 ± 1.2^abc^	37.6 ± 1.6^cd^	6.7 ± 1.1^hijk^	20.6 ± 1.9^jkl^	0.015 ± 0.000^ef^	51.8 ± 1.1^ijk^	2.2	0.966
	Ripe	Boiled	34.3 ± 4.5^ij^	29.9 ± 0.5^cde^	4.2 ± 0.5^gh^	0.3 ± 0.0^m^	6.5 ± 0.3^ijk^	99.1 ± 0.0^a^	0.102 ± 0.000^a^	104.7 ± 0.0^a^	2.6	0.996
Guineo	Unripe	Raw	67.5 ± 1.4^cde^	1.6 ± 0.1^l^	13.5 ± 0.1^bcd^	52.4 ± 0.0^bc^	11.9 ± 0.0^defg^	11.9 ± 0.0^m^	0.012 ± 0.000^f^	47.1 ± 0.0^l^	1.8	0.955
	Unripe	Boiled	64.6 ± 0.3^def^	19.7 ± 0.2^efg^	26.1 ± 1.7^ab^	18.9 ± 1.5^fg^	14.2 ± 0.5^cdef^	68.4 ± 0.0^efg^	0.045 ± 0.013^abcde^	80.4 ± 1.5^def^	1.8	0.996
	Ripe	Raw	23.2 ± 0.0^j^	4.4 ± 0.1^jk^	13.2 ± 1.1^cdef^	5.6 ± 0.9^hi^	2.0 ± 0.0^k^	63.1 ± 0.7^fgh^	0.024 ± 0.004^bcdef^	75.5 ± 0.4^efg^	5.9	0.956
	Ripe	Boiled	16.8 ± 0.1^j^	15.6 ± 1.0^fgh^	0.7 ± 1.0^h^	0.4 ± 0.0^kl^	3.1 ± 0.0^jk^	97.4 ± 0.0^ab^	0.092 ± 0.003^a^	102.9 ± 0.2^ab^	7.1	0.999
**Statistical significance of the sources of variation SS**												
Local name of cultivar (C)			<0.001 ***	<0.001 ***	<0.001 ***	<0.001 ***	<0.001 ***	<0.001 ***	0.3754 ns	<0.001 ***		
Ripening stage (R)			<0.001 ***	<0.001 ***	<0.001 ***	<0.001 ***	<0.001 ***	<0.001 ***	0.0018 **	<0.001 ***		
Processing (P)			<0.001 ***	<0.001 ***	0.0261 *	<0.001 ***	<0.001 ***	<0.001 ***	<0.001 ***	<0.001 ***		
Interaction (CxRxP)			<0.001 ***	<0.001 ***	<0.001 ***	<0.001 ***	<0.001 ***	<0.001 ***	0.0477 *	<0.001 ***		

Values are means ± SD (*n* = 2 analytical replicates). Means within each column followed by different superscript letters differ significantly (α = 0.05). 16 cultivar (C) × ripening stage (R) × processing (P) combinations were compared by the Tukey–Kramer test on rank-transformed data (rank transform, RT), and the main effects of C, R and P and the C × R × P interaction were tested by aligned rank transform ANOVA (ART). Significance codes: ** *p* < 0.01; * *p* < 0.05; ns, not significant. TS, total starch; RDS, rapidly digestible starch; SDS, slowly digestible starch; RS, resistant starch; HI, hydrolysis index; C∞, equilibrium concentration of hydrolysed starch; k, kinetic constant of starch hydrolysis (min^−1^); eGI, estimated glycemic index.

**Table 3:** Organic acid and sugar content in flours from different cultivars, stages of ripening and processing methods.

Local name of Cultivar	Ripening stage	Processing	Organic acids				Sugars				Total Sugar (mg/g, db)
			Oxalic (mg/g, db)	Citric (mg/g, db)	Malic (mg/g, db)	Succinic (mg/g, db)	Raffinose (mg/g, db)	Sucrose (mg/g, db)	Glucose (mg/g, db)	Fructose (mg/g, db)	
Dominico Harton	Unripe	Raw	0.12 ± 0.00^p^	11.3 ± 0.1^lm^	3.4 ± 0.1^no^	1.7 ± 0.1^kl^	0.4 ± 0.0^l^	0.9 ± 0.0^n^	10.5 ± 0.1^lm^	12.4 ± 0.1^mn^	24.2 ± 0.2^no^
	Unripe	Boiled	0.97 ± 0.00^b^	10.8 ± 0.1^mn^	2.8 ± 0.0^o^	1.0 ± 0.0^m^	1.2 ± 0.0^j^	11.7 ± 0.1^ij^	2.8 ± 0.0^o^	4.1 ± 0.0^o^	19.8 ± 0.1^o^
	Ripe	Raw	0.18 ± 0.00^m^	23.2 ± 0.3^cd^	20.6 ± 0.0^de^	5.6 ± 0.1^bc^	4.7 ± 0.0^de^	7.9 ± 0.0^jk^	270.0 ± 2.3^ab^	280.9 ± 2.2^bc^	563.5 ± 4.6^cd^
	Ripe	Boiled	0.75 ± 0.00^d^	22.4 ± 0.0^de^	8.8 ± 0.0^hi^	4.3 ± 0.0^cd^	5.2 ± 0.0^bc^	343.5 ± 0.8^ab^	78.2 ± 0.3^fg^	62.4 ± 0.0^gh^	489.3 ± 1.1^ef^
Cachaco	Unripe	Raw	0.16 ± 0.00^n^	9.1 ± 0.1^o^	8.5 ± 0.2^ij^	3.9 ± 0.1^ef^	0.7 ± 0.1^k^	1.8 ± 0.1^lm^	37.3 ± 0.4^hi^	43.4 ± 0.6^hi^	83.3 ± 1.2^jk^
	Unripe	Boiled	0.60 ± 0.01^f^	10.5 ± 0.2^no^	6.2 ± 0.1^lm^	2.3 ± 0.1^jk^	1.3 ± 0.0^hi^	104.4 ± 0.6^de^	21.6 ± 0.1^jk^	20.7 ± 0.3^kl^	148.1 ± 0.4^hi^
	Ripe	Raw	0.22 ± 0.02^l^	23.3 ± 0.1^bc^	22.2 ± 0.2^cd^	4.3 ± 0.1^cd^	1.8 ± 0.0^gh^	7.3 ± 0.0^kl^	228.8 ± 0.8^cd^	244.8 ± 0.4^cd^	482.5 ± 1.2^fg^
	Ripe	Boiled	0.53 ± 0.00^g^	22.2 ± 0.2^ef^	9.4 ± 0.0^gh^	2.7 ± 0.1^ij^	4.0 ± 0.1^ef^	253.1 ± 0.4^cd^	88.4 ± 0.6^ef^	79.0 ± 0.3^ef^	424.6 ± 1.3^gh^
Guayabo	Unripe	Raw	0.13 ± 0.00^o^	14.6 ± 0.1^jk^	7.1 ± 0.0^kl^	3.1 ± 0.1^gh^	0.5 ± 0.0^kl^	1.1 ± 0.0^mn^	17.0 ± 0.2^kl^	22.4 ± 0.2^jk^	41.1 ± 0.3^lm^
	Unripe	Boiled	0.66 ± 0.00^e^	13.0 ± 0.1^kl^	4.7 ± 0.0^mn^	1.2 ± 0.1^lm^	0.7 ± 0.0^k^	17.9 ± 0.1^gh^	3.9 ± 0.1^no^	6.6 ± 0.1^no^	29.2 ± 0.2^mn^
	Ripe	Raw	0.37 ± 0.01^h^	29.6 ± 0.1^a^	29.4 ± 0.1^ab^	4.1 ± 0.0^de^	2.3 ± 0.0^fg^	12.5 ± 0.0^hi^	266.5 ± 1.2^bc^	289.2 ± 1.1^ab^	570.5 ± 2.2^bc^
	Ripe	Boiled	0.26 ± 0.00^k^	25.9 ± 0.0^ab^	13.5 ± 0.2^ef^	2.9 ± 0.1^hi^	5.1 ± 0.0^cd^	392.6 ± 0.8^a^	74.5 ± 0.0^gh^	63.5 ± 0.0^fg^	535.8 ± 0.9^de^
Guineo	Unripe	Raw	0.28 ± 0.00^j^	19.4 ± 0.2^gh^	12.0 ± 0.2^fg^	3.6 ± 0.1^fg^	1.3 ± 0.0^ij^	19.2 ± 0.3^fg^	34.2 ± 0.2^ij^	42.0 ± 0.3^ij^	96.6 ± 0.8^ij^
	Unripe	Boiled	1.25 ± 0.02^a^	16.7 ± 0.3^hi^	7.7 ± 0.1^jk^	1.6 ± 0.0^kl^	1.1 ± 0.0^j^	36.4 ± 0.5^ef^	10.2 ± 0.1^mn^	14.1 ± 0.2^lm^	61.9 ± 0.8^kl^
	Ripe	Raw	0.32 ± 0.04^i^	19.7 ± 0.1^fg^	38.8 ± 0.3^a^	9.8 ± 0.1^a^	10.4 ± 0.0^a^	107.3 ± 8.7^de^	309.2 ± 4.1^a^	334.8 ± 4.7^a^	761.7 ± 0.0^a^
	Ripe	Boiled	0.96 ± 0.01^c^	16.4 ± 0.0^ij^	23.4 ± 0.2^bc^	7.6 ± 0.0^ab^	8.9 ± 0.0^ab^	0.9 ± 0.0^n^	141.4 ± 0.0^de^	143.8 ± 0.4^de^	628.6 ± 0.8^ab^
**Statistical significance of the sources of variation**											
Local name of cultivar (C)			<0.001 ***	<0.001 ***	<0.001 ***	<0.001 ***	<0.001 ***	<0.001 ***	<0.001 ***	<0.001 ***	<0.001 ***
Ripening stage (R)			<0.001 ***	<0.001 ***	<0.001 ***	<0.001 ***	<0.001 ***	<0.001 ***	<0.001 ***	<0.001 ***	<0.001 ***
Processing (P)			<0.001 ***	<0.001 ***	<0.001 ***	<0.001 ***	<0.001 ***	<0.001 ***	<0.001 ***	<0.001 ***	<0.001 ***
Interaction (CxRxP)			<0.001 ***	<0.001 ***	<0.001 ***	<0.001 ***	<0.001 ***	<0.001 ***	<0.001 ***	<0.001 ***	<0.001 ***

Values are means ± SD (*n* = 2 analytical replicates). Means within each column followed by different superscript letters differ significantly (α = 0.05). 16 cultivar (C) × ripening stage (R) × processing (P) combinations were compared by the Tukey–Kramer test on rank-transformed data (rank transform, RT), and the main effects of C, R and P and the C × R × P interaction were tested by aligned rank transform ANOVA (ART). Total sugars = raffinose + sucrose + glucose + fructose. Significance codes: *** *p* < 0.001; ** *p* < 0.01; * *p* < 0.05; ns, not significant.

**Table 4:** Pasting properties, solubility, swelling power, onset temperature (To) and gelatinization enthalpy (∆H) of flours from different cultivars, stages of ripening and processing methods.

Local name of Cultivar	Ripening stage	Processing	Pasting temperature (°C)	Peak viscosity (cP)	Trough (cP)	Final viscosity (cP)	Breakdown (cP)	Setback (cP)	Solubility (%, db)	Swelling power (g/g)	To (°C)	ΔH (J/g)
Dominico Harton	Unripe	Raw	74.1 ± 0.4^abc^	2297.0 ± 19.8^a^	1601.0 ± 24.0^ab^	2083.0 ± 32.5^a^	696.0 ± 4.2^a^	482.0 ± 8.5^a^	15.1 ± 1.2^ij^	11.7 ± 0.0^def^	61.0 ± 0.6^de^	12.2 ± 0.8^a^
	Unripe	Boiled	60.0 ± 1.1^def^	987.5 ± 44.5^de^	880.5 ± 23.3^ef^	1203.5 ± 16.3^ef^	107.0 ± 21.2^de^	323.0 ± 7.1^bc^	17.4 ± 0.4^hi^	12.3 ± 0.6^cde^	ND	ND^d^
	Ripe	Raw	ND	ND	ND	161.5 ± 6.4^kl^	ND	ND	55.8 ± 0.2^abc^	15.3 ± 0.8^ab^	67.9 ± 1.3^ab^	5.0 ± 0.5^bc^
	Ripe	Boiled	ND	ND	ND	94.5 ± 16.3^lm^	ND	ND	28.8 ± 0.9^efg^	8.4 ± 0.3^h^	ND	ND^d^
Cachaco	Unripe	Raw	75.9 ± 0.2^ab^	1636.5 ± 47.4^bc^	1228.0 ± 8.5^bc^	1493.5 ± 16.3^cd^	408.5 ± 55.9^ab^	265.5 ± 7.8^bc^	11.5 ± 0.1^k^	9.0 ± 0.5^gh^	60.4 ± 0.5^e^	11.2 ± 0.4^ab^
	Unripe	Boiled	58.3 ± 2.5^ef^	875.0 ± 17.0^f^	527.0 ± 1.4^g^	695.0 ± 12.7^gh^	348.0 ± 15.6^bc^	168.0 ± 14.1^c^	26.1 ± 1.8^fgh^	16.9 ± 0.6^a^	ND	ND^d^
	Ripe	Raw	ND	ND	ND	355.5 ± 6.4^hi^	ND	ND	33.8 ± 1.3^de^	10.7 ± 1.1^fg^	64.0 ± 0.0^bcd^	4.1 ± 0.0^c^
	Ripe	Boiled	ND	ND	ND	248.0 ± 7.1^ij^	ND	ND	44.6 ± 3.0^bcd^	14.0 ± 0.4^abc^	ND	ND^d^
Guayabo	Unripe	Raw	79.0 ± 0.2^a^	1823.0 ± 24.0^ab^	1672.5 ± 10.6^a^	2018.0 ± 19.8^ab^	150.5 ± 13.4^cde^	345.5 ± 9.2^ab^	13.3 ± 0.4^jk^	10.6 ± 0.5^fg^	64.8 ± 0.4^abc^	13.1 ± 1.1^a^
	Unripe	Boiled	61.2 ± 0.8^cde^	1154.0 ± 11.3^cd^	999.0 ± 4.2^de^	1350.0 ± 15.6^de^	155.0 ± 15.6^cd^	351.0 ± 19.8^ab^	18.2 ± 1.0^ghi^	12.8 ± 0.1^bcd^	ND	ND^d^
	Ripe	Raw	ND	ND	ND	165.0 ± 1.4^kl^	ND	ND	43.3 ± 2.1^cd^	10.6 ± 0.6^fg^	69.4 ± 0.1^a^	4.5 ± 0.4^bc^
	Ripe	Boiled	ND	ND	ND	84.0 ± 4.2^m^	ND	ND	33.3 ± 1.6^def^	10.8 ± 0.6^efg^	ND	ND^d^
Guineo	Unripe	Raw	70.6 ± 0.1^bcd^	1829.5 ± 4.9^ab^	1207.5 ± 12.0^cd^	1560.5 ± 30.4^bc^	622.0 ± 17.0^ab^	353.0 ± 42.4^ab^	12.8 ± 0.8^jk^	9.4 ± 0.4^gh^	62.3 ± 1.3^cde^	11.9 ± 0.8^a^
	Unripe	Boiled	54.5 ± 0.6^f^	898.5 ± 14.8^ef^	796.5 ± 34.6^fg^	1037.0 ± 53.7^fg^	102.0 ± 19.8^e^	240.5 ± 19.1^c^	17.5 ± 0.1^hi^	12.4 ± 0.6^cd^	ND	ND^d^
	Ripe	Raw	ND	ND	ND	19.5 ± 9.2^m^	ND	ND	76.5 ± 0.4^a^	15.6 ± 0.3^a^	ND	ND^d^
	Ripe	Boiled	ND	ND	ND	191.0 ± 4.2^jk^	ND	ND	65.8 ± 0.6^ab^	14.5 ± 0.3^abc^	ND	ND^d^

**Statistical significance of the sources of variation**												
Local name of cultivar (C)			0.0013 **	< 0.001 ***	< 0.001 ***	< 0.001 ***	0.0117 *	< 0.001 ***	< 0.001 ***	< 0.001 ***	0.0011 **	< 0.001 ***
Ripening stage (R)			–	< 0.001 ***	< 0.001 ***	< 0.001 ***	–	–	< 0.001 ***	0.0074 **	0.0049 **	< 0.001 ***
Processing (P)			0.0011 **	< 0.001 ***	< 0.001 ***	< 0.001 ***	0.0011 **	0.0011 **	0.0063 **	< 0.001 ***	–	< 0.001 ***
Interaction (CxRxP)			–	< 0.001 ***	< 0.001 ***	< 0.001 ***	–	–	< 0.001 ***	0.0029 **	–	< 0.001 ***

Values are means ± SD (*n* = 2 analytical replicates). Means within each column followed by different superscript letters differ significantly (α = 0.05). 16 cultivar (C) × ripening stage (R) × processing (P) combinations were compared by the Tukey–Kramer test on rank-transformed data (rank transform, RT), and the main effects of C, R and P and the C × R × P interaction were tested by aligned rank transform ANOVA (ART). ND not detected (no pasting peak or no gelatinization endotherm). Peak viscosity, trough and ΔH values of ND were treated as zero; pasting temperature and To values of ND were excluded from the analysis. Breakdown and setback could not be determined (ND) in ripe samples because no viscosity peak was observed. For pasting temperature, breakdown and setback, the effects of C and P were tested in unripe samples (complete C × P design); for To, the effects of C and R were tested in raw samples of Dominico Hartón, Cachaco and Guayabo (complete C × R design); —, effect not estimable. Significance codes: *** *p* < 0.001; ** *p* < 0.01; * *p* < 0.05; ns, not significant. To, onset gelatinization temperature; ΔH, gelatinization enthalpy; db, dry basis.

Results are expressed as mean ± standard deviation (SD) and means within the same column sharing at least one lowercase superscript letter do not differ significantly (α = 0.05). Regarding pasting and thermal properties, treatments lacking a peak viscosity or a gelatinization endotherm were reported as ND (not detected) and excluded from the analysis. Consequently, the effects of C and P on pasting temperature, peak viscosity, trough viscosity, breakdown, and setback were evaluated in unripe samples (C x P), while the effects of C and R on To and ΔH were evaluated in raw samples of Dominico Hartón, Cachaco, and Guayabo (C x R).

Additionally, principal component analysis (PCA) was performed on the standardized treatment means (C x R x P; correlation matrix), retaining components with eigenvalues >1. Pearson correlation coefficients (r) between variables were calculated using the same treatment means and were considered significant at *p* < 0.05.

All statistical analyses were performed using JMP Pro 14 (SAS Institute Inc., Cary, NC, USA).

## 3 Results and discussion

### 3.1 Effect of ripening and cooking on dry matter and carotenoid content in pulp

The results in Table 1 indicate a decrease in dry matter content during cooking and fruit ripening. In the first case, this is due to leaching and hydration during cooking, and in the second case, is mainly associated with starch degradation, respiratory losses (Cordenunsi-Lysenko et al., 2019), and changes in tissue water distribution going with the accumulation of soluble sugars in the pulp (Moreno et al., 2021). Furthermore, the interaction between cultivar, ripening stage, and processing showed a highly significant effect (*p* < 0.0001) on dry matter content.

The carotenoid pigments identified in higher proportion in the evaluated cooking bananas were t-βC, αC followed by Lutein and in lower proportion some isomers such as 9-cis-βC, 13-cis-βC and 15-cis-βC, these pigments present different chemical structures, therefore, they present different provitamin A activities (Ekesa et al., 2015). Independent spectrophotometric (UV) and HPLC-DAD quantifications were highly concordant across all cultivars, ripening stages, and processing conditions evaluated, over a carotenoid concentration range of 109.9–1666.7 μg/100 g FW (Pearson *r* = 0.977, *p* < 0.0001), confirming the reliability of the extracted carotenoid fraction. The average total carotenoids ranged from 109.9 μg/100 g f.w. (Guineo, Unripe+Raw) to 1666.6 μg/100 g f.w (Dominico Harton, Unripe+Raw). The concentration of t-βC and αC accounts for more than 90% of the carotenoid pVACs, with t-βC being the carotenoid with the highest concentration in most of the genotypes analyzed except for Guineo where the main carotenoid is αC (Table 1). High levels of provitamin compounds were found in cooking bananas with a visually more yellowish (somewhat orange) flesh coloration, as is the case of Dominico Harton (Unripe+Raw), compared to lighter colored genotypes that usually have lower amounts of carotenoids, mainly pVACs, as is the case of Guineo, which presented higher concentration of Lutein compared to the other cultivars (Table 1). Lutein has antioxidant activity (Miller et al., 1996) and has been associated with a decreased risk of macular degeneration (IOM, 2000). Lutein content increased with the degree of ripening and cooking in all samples evaluated. Ripening stage had no significant effect on total carotene content (*p* = 0.2088), but processing and cultivar did affect carotene content (*p* < 0.001).

According to Pearson's correlations between t-βC and αC contents, a highly significant correlation was obtained (0.7087, *p* < 0. 0001). These correlations are higher when related by cultivar and coefficients of 0.999 (*p* < 0.0001), 0.9417 (*p* < 0.0001), 0.9863 (*p* < 0.0001) and 0.9204 (*p* < 0.0001) were obtained for Dominico Harton, Cachaco, Guineo and Guayabo, respectively. The high degree of correlation between individual carotenoids is probably because these compounds share a common biosynthetic pathway. The substantial phenotypic variation in pVAC content observed among the four cultivars is consistent with the existence of a genetic basis for this trait, as documented in broader germplasm studies (Amah et al., 2019; Englberger et al., 2003), and supports the rationale for including provitamin A carotenoid content as a selection criterion in cooking banana breeding programs.

The Guineo cultivar showed an increase in total carotenoid content during ripening. These results are consistent with those reported by Ngoh Newilah et al. (2008) and Ekesa et al. (2012), who observed a significant increase in carotenoids with provitamin A activity in different banana cultivars from Cameroon and East Africa as ripening progressed. Similarly, Kato et al. (2004) documented an accumulation of carotenoids in citrus fruits during ripening, indicating that this process is tightly regulated by the coordinated expression of genes involved in their biosynthesis. By analogy with molecular mechanisms described in tomato, including upregulation of PSY (phytoene synthase) and PDS (phytoene desaturase) and downregulation of LCYb (lycopene β-cyclase) and LCYe (lycopene ε-cyclase) during ripening (Isaacson et al., 2002; Ronen et al., 1999), similar regulatory processes may contribute to carotenoid accumulation in Guineo; however, no gene expression analysis was performed in this study, so such mechanistic interpretations remain speculative for the cultivars evaluated. In the case of Dominico Harton and Guayabo, a statistically significant reduction (*p* < 0.05) in total carotenoids content from unripe to ripe was presented.

Dominico Harton presented the highest t-βC content (926.1 μg/ 100 g f.w., Unripe+Raw), this genotype is widely used in Colombia, followed by Cachaco (531.3 μg/ 100 g f.w., Unripe+Raw). In the case of Dominico Harton, Cachaco and Guayabo there was a decrease in pVACs during ripening, probably due to degradation, oxidation and/or isomerization processes of these bioactive forms (Schieber & Carle, 2005).

Dominico Harton and Cachaco presented a decrease in t-βC and αC, during ripening and thermal processing, except for Guineo and Guayabo (Ripe+Boiled), this decrease could be due to their degradation, which could stimulate the synthesis of other carotenoid isomers during post-harvest ripening; this phenomenon could have been enhanced by the processing conditions (storage and cooking). The content of t-βC isomers increased with ripening stage and cooking process, although cis-carotenoids are usually present in tissues, they tend to be formed by non-enzymatic isomerization events. In this study, isomers of t-βC were identified as 15-cis, 13-cis and 9 cis β-carotene (Table 1), which are degradation products of t-βC. For Dominico Harton, Cachaco and Guayabo. Borges et al. (2019) found the concentration of the isomers increased with the cooking process in stages unripe and ripe compared to the results in fresh, this similar behavior. In the case of Guineo unripe, no isomerization was detected both in raw and cooked, only isomerization was presented in Ripe+Boiled. However, it should be noted that, although the proportion of cis-isomers is low, the consequences on sensory and nutritional properties might be important because they have lower antioxidant properties than t-βC (Englberger et al., 2003).

Thermal processing had an impact on pVACs in all genotypes evaluated. The study of carotenoid retention after thermal processing generates much interest, both at industrial and domestic levels, since losses of these nutrients can be generated depending on the method used (Rodriguez-Amaya et al., 2006), although the interpretation and comparison of these data is often difficult due to different processing conditions and different extraction methods (Ekesa et al., 2012). However, Ekesa et al. (2012) reported an increase of total pVACs in the genotype ‘Musilongo’ after boiling treatment compared to raw analysis, this result has been previously found by Borges et al. (2019) in the genotypes ‘D'Angola’ and ‘Pelipita’. In this study, similar results were obtained for all genotypes evaluated in the ripe state. Rodriguez-Amaya et al. (2006), report that after cooking, pigments can be extracted more effectively from the food matrix by the solvent; in addition, the enzymatic degradation of structural carbohydrates occurs during the ripening of bananas and plantains (Kojima et al., 1994), which may also contribute to the increased extraction of carotenoids by the solvent after cooking. In all cooked samples, there was a loss of moisture compared to the raw sample, which may also explain the increase in carotenoids in the cooked samples at stage ripening. However, this may also depend on the cooking method, as longer cooking duration and high temperatures destroy carotenoids. In the case of Dominico Harton, Cachaco and Guayabo in unripe and after cooking, they showed a decrease in carotenoid content compared to the same stage in fresh. These differences found in carotenoid content under the same heat treatment, among the cultivars evaluated, may be the result of inherent differences between genotypes, also the cultivar presented a highly significant effect (*p* < 0.001) on all carotenoid pigments and dry matter, this behavior was similar for the interaction (CxRxP).

#### 3.1.1 Contribution to vitamin A requirements

RAE was calculated based on the contribution of trans-β-carotene, α-carotene, and β-carotene isomers, with trans-β-carotene acting as the primary determinant due to its higher bioconversion efficiency to retinol. So, Dominico Harton exhibited the highest RAE (98.8 μg/100 g fw, Unripe+Raw), followed by Cachaco (47.5 μg/100 g fw, Unripe+Raw), while Guineo showed the lowest values (4.0 μg/100 g fw, Unripe+Raw). Based on the threshold proposed by Englberger et al. (2010), Dominico Harton can be considered a good source of provitamin A, although its nutritional potential is strongly affected by processing.

Thermal treatment reduced RAE when carotenoid degradation exceeded matrix disruption, as observed in Dominico Harton, where only ∼42% of provitamin A carotenoids were retained after cooking at the unripe stage. This reduction is consistent with the known thermal sensitivity of β-carotene, including oxidative degradation and isomerization (Rodriguez-Amaya, 2006). Thermal disruption of the cell wall matrix enhanced carotenoid extractability by organic solvents, which may contribute to improved bioaccessibility, although this was not directly measured in the present study (Palmero et al., 2013). Thus, the net effect of processing depends on the balance between degradation kinetics and matrix-driven carotenoid release. Ripening influenced carotenoid functionality primarily through compositional changes rather than total accumulation. Although total carotene content was not significantly affected (*p* = 0.2088) by ripening, provitamin A-active carotenoids (pVACs) showed a significant effect (*p* = 0.0096), indicating selective biosynthesis and/or transformation of carotenoid fractions during ripening. Consequently, the RAE value was highly significantly affected by the (CxRxP) interaction, confirming its sensitivity to genotype, ripening, and processing conditions.

From a nutritional perspective, the evaluated cultivars contributed between ∼2.8–24.9% of the daily recommended intake (DRI) for children (1–3 years, 400 RAE μg/day, FAO/WHO, 2002) under typical consumption scenarios (250 g, Unripe+Bolied), a portion size consistent with the consumption level applied for this age group by Englberger et al. (2003) using the same DRI reference. This contribution increased to ∼16.9–20.9% at the Ripe+Bolied. These values are consistent with previous reports indicating that certain plantain cultivars can provide a substantial proportion of vitamin A requirements (Englberger et al., 2003; Fungo & Pillay, 2011).

However, as plantains are typically cooked, their effective contribution to dietary vitamin A depends on processing-induced changes in carotenoid stability and bioavailability (Ruel, 2001). Overall, these results demonstrate that RAE is governed by the interaction between carotenoid composition and thermal stability. Consequently, genotypes with high total carotenoids do not necessarily exhibit high provitamin A activity. Finding cultivars with favorable carotenoid profiles and improved stability under processing conditions represents a key strategy for enhancing dietary vitamin A intake and supporting biofortification and food security initiatives.

### 3.2 Total starch, in vitro digestion properties, amylose content and kinetic parameters and estimated glycemic index of flour from different cultivars

Starch is the main component of unripe cooking bananas and undergoes significant degradation during the ripening process. Significant differences (*p* < 0.01) in initial starch content were observed among the four studied varieties. Overall, the behavior of starch content was as follows: Unripe+Raw > Unripe+Boiled > Ripe+Raw > Ripe+Boiled (Table 2). Starch content decreased during ripening, a result of starch hydrolysis into simple sugars due to various enzymes such as amylases and amyloglucosidase; furthermore, different rates of hydrolysis were observed, a behavior also noted by Moreno et al. (2021). Additionally, a reduction in starch content was observed due to cooking, which causes partial gelatinization of starch. During gelatinization, starch granules swell and absorb water potentially allowing amylose to be released into the aqueous phase (Wang & Copeland, 2013).

The cultivar (C), ripeness stage (R), processing (P), and their interaction (CxRxP) had a highly significant effect (*p* ≤0.001) on starch content (Table 2).

The rapidly digestible starch (RDS), slow digestible starch (SDS), and resistant starch (RS) fractions in the flours ranged from 0.9% to 48.0%, 0.7% to 40.4%, and 0.3% to 71.1%, respectively, showing differences in starch degradation systems among the evaluated cultivars. The samples (Unripe+Raw) showed high RS content, indicating that banana flour is a good source of resistant starch. Dominico Harton (Unripe+Raw) had the highest RS content, implying a high resistance to enzymatic digestion. The morphology of starch granules, the surface area of the starch granules, the pores present on the surface of the starch granules, and the crystalline structure are key factors affecting the digestibility properties of starch (Chang et al., 2022). Additionally, components such as proteins, pectin, and polyphenols present in the flours can limit enzymatic activity by reducing the contact between enzymes and starch (Bi et al., 2017). The cultivars in their unripe and raw state showed high levels of native starch, which is minimally susceptible to hydrolysis (Gonzalez-Soto et al., 2007). This translates into a high content of resistant starch (RS) and confirms the potential of plantain flour as a significant source of this fraction. Ripening and cooking reduced the RS content. Heat treatment induces starch gelatinization, with loss of crystalline structure and increased digestibility (Wang & Copeland, 2013). Consequently, the digestibility of the Ripe+Boiled flours increased significantly (*p* < 0.001), and the Unripe+Boiled and Ripe+Boiled samples showed higher concentrations of rapidly digestible starch (RDS), saying partial or total degradation of the resistant starch (RS). Approximately 95% of the RS in Ripe+Boiled was transformed into RDS and SDS fractions, consistent with the findings reported by Cahyana et al. (2019). In the unripe state, Guineo retained the highest proportion of RS after boiling (∼36%; from 52.4% Unripe+Raw to 18.9% Unripe+Boiled), while Guayabo showed the greatest proportional reduction, retaining only ∼22% of its original RS content (from 63.3% Unripe+Raw to 14.1% Unripe+Boiled). Taken together, these findings indicate that the stage of ripeness and heat treatment determine the distribution of starch fractions and, so, the potential glycemic and prebiotic impact of the flours. Therefore, using it in its raw state or applying hydrothermal strategies aimed at modulating the SDS fraction could improve its nutritional functionality. Furthermore, the interaction (C × R × P) showed a highly significant effect (*p* < 0.001) on the resistant starch (RS), slow-digesting starch (SDS), and rapidly digestible starch (RDS) content (Table 2). Unripe plantain flour was characterized by its high RS and SDS content, fractions associated with a lower glycemic response.

Amylose content is a key determinant of starch's physicochemical properties, including its rheological behavior, gelatinization, retrogradation, and paste formation (Baik & Lee, 2003). In this study, amylose was highest in unripe samples and decreased significantly during ripening because of starch conversion to simple sugars. For example, in the Dominico Harton cultivar, amylose content decreased from 18.7% (Unripe+Raw) to 6.7% (Ripe+Raw), a trend consistent across all evaluated genotypes. Thermal processing also significantly influenced amylose content. In Guayabo and Guineo, the boiled samples showed higher values than the raw samples; for the Guayabo variety, the amylose content increased from 15.5% (Unripe+Raw) to 19.1% (Unripe+Boiled). This increase could be attributed to possible effects resulting from the loss of dry matter*.* During gelatinization, starch granules swell and absorb water, potentially allowing amylose to be released into the aqueous phase (Wang & Copeland, 2013); however, starch release into the cooking water was not directly quantified in this study, and the observed reduction in the flour's starch content after boiling is attributed to the combined effects of gelatinization, partial solubilization, and dry matter dilution.

Resistant starch is predominantly derived from the retrograded fraction of amylose, which forms linear alpha-1,4-D-glucan structures resistant to enzymatic digestion (Fuentes-Zaragoza et al., 2010; Tharanathan, 2002)*.* RS content is positively correlated with the proportion of amylose present in the starch, as reported by Gao et al. (2016). Consistent with these reports, the present study saw a significant linear correlation between amylose content and RS in raw unripe and ripe cooking banana flours (R^2^ = 0.901; *p* < 0.01; Fig. S1a). However, when the data for the flours after cooking were also considered, this relationship ceased to be significant (R^2^ = 0.1267; *p* > 0.05, Fig. S1b). This result suggests that thermal processing alters the starch structure, modulating its digestibility and reducing the intrinsic compositional influence of amylose on the glycemic index. The high coefficient of determination in raw samples suggests that amylose content can be considered a reliable predictor of RS in these materials. This finding has technological and nutritional relevance, given that RS is associated with a lower glycemic index and prebiotic effects, potentially contributing to metabolic and intestinal health.

To simplify eGI estimation, we derived an empirical equation using H80exp as a single time-point predictor: eGI = 38.59 + 0.63 H80 (R2 = 0.991, *p* < 0.01). While this approach differs methodologically from the area under the curve based hydrolysis index (HI) of Goñi et al. (1997), the strong predictive relationship observed indicates that H80 adequately captures the digestibility information required to estimate eGI in these banana flour matrices, without requiring the full kinetic characterization.

The estimated glycemic index (eGI) was below 60 for the Unripe+Raw and Ripe+Raw samples, except for the Guineo variety (Ripe+Raw), whose GI was 75. Compared to international GI tables (Foster-Powell et al., 2002), the reported GI values were 57 for unripe plantains from Ghana, 54 for unripe bananas from New Zealand, and 69 for overripe bananas from the United States, using white bread as a reference, which is consistent with the results of this study. In contrast, the Unripe+Boiled and Ripe+Boiled samples exceeded 80, even reaching over 100 in varieties such as Guayabo and Guineo. This increase is explained by the combined effect of ripeness and cooking: the increase in sugars in the ripe fruit and the reduction of resistant starch during cooking facilitate starch digestion. The increase in the eGI after cooking was positively correlated with rapidly digestible starch (RDS) and the kinetic constant k, and inversely correlated with resistant starch (RS), confirming that ripening and heat treatment interact to modulate digestibility and the glycemic response. The glycemic response depends on structural factors of starch, such as granule morphology, the amylose-amylopectin ratio, branching, and molecular structure (Fuentes-Zaragoza et al., 2010). Furthermore, fitting the experimental data to the first-order exponential model yielded high coefficients of determination (R^2^ = 0.895–1.000) and low RMSE values (0.4–7.6%, db), confirming the suitability of the kinetic model.

### 3.3 Adjustment of digestion kinetics according to the logarithm of slope (LOS) model

The results of the in vitro digestibility kinetics are presented in Fig. 1 for the four varieties evaluated. Digestion rates ranged from 19.9% to 25.6% in the Unripe+Raw samples and from 31.6% to 75.8% in the Ripe+Raw samples. In the case of the cooked samples, the rates ranged from 70.8% to 97% for the unripe samples, and from 97.4% to 99.2% for the ripe samples, during an enzymatic hydrolysis period of 120 min. The Unripe+Raw flour showed high resistance to digestion. In general, digestion followed the following trend: Ripe+Boiled > Unripe+Boiled > Ripe+Raw > Unripe+Raw. This confirms that heat treatment has a greater influence on digestion than maturation. The linearity observed in the LOS plots indicated a single-phase digestion process; however, some Unripe+Raw samples showed slight deviations, suggesting greater structural heterogeneity and the coexistence of fractions with different enzymatic susceptibility.

**Fig. 1:**
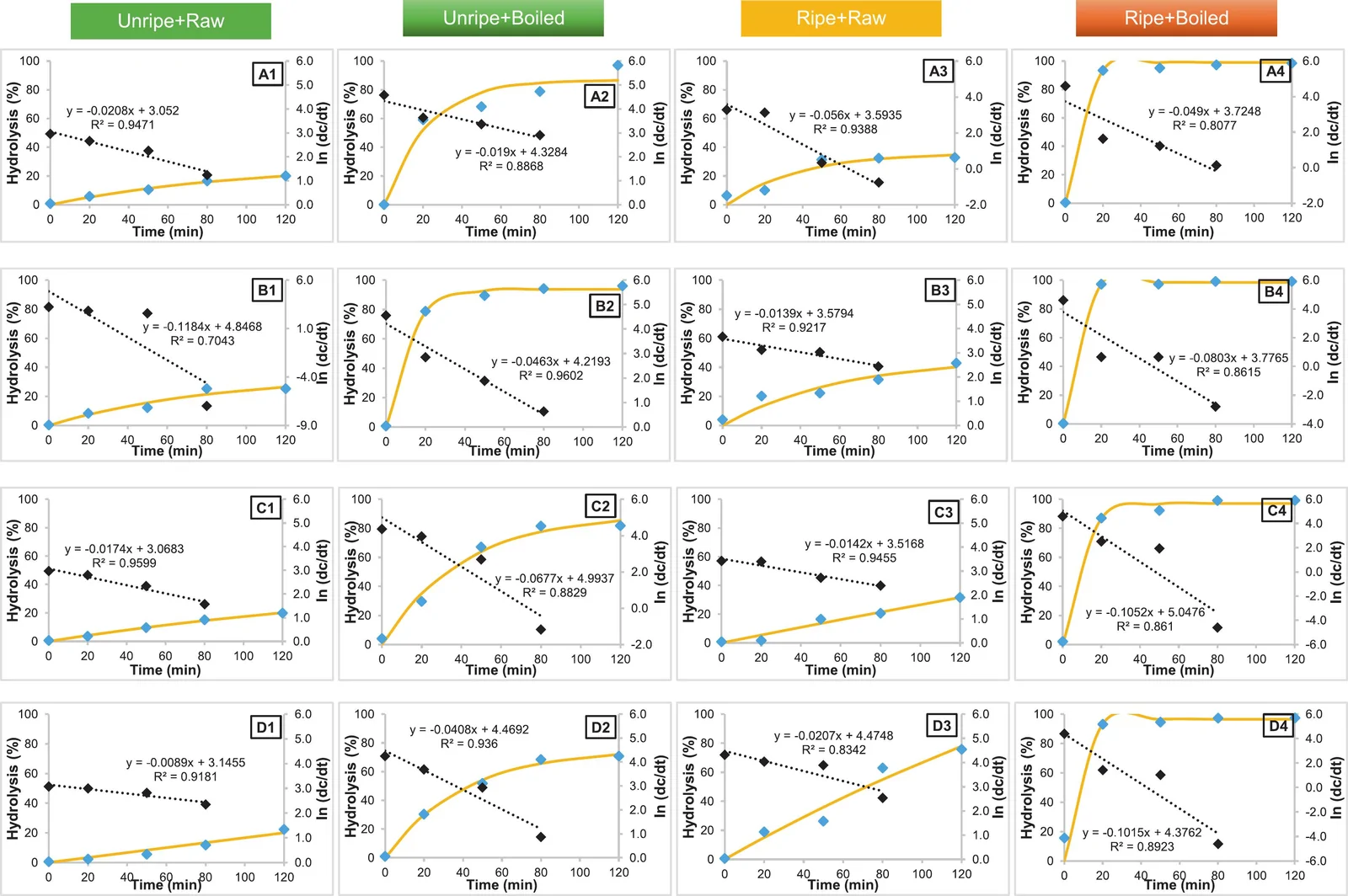
Hydrolysis kinetics and LOS plots for the flours tested. Experimental data; Model fit; LOS plot data; Linear (LOS plot data). Dominico Harton (A); Cachaco (B); Guayabo (C); Guineo (D). A1-D1: Unripe+Raw; A2-D2: Unripe+Boiled; A3-D3: Ripe+Raw; A4-D4: Ripe+Boiled.

The C80 parameter and the hydrolysis rate constant (k) increased significantly after cooking, especially in the Ripe+Boiled samples. Boiled ripe Guayabo showed the highest values for k (0.102 min^−1^) and C80 (99.14%), showing almost complete hydrolysis. These results show that both ripening and cooking significantly influence digestibility. In the case of ripening, this effect can be attributed to the partial degradation of starch and the decrease in structural order, which facilitates enzymatic access. Cooking, on the other hand, promotes starch gelatinization and the disruption of the crystalline structure, increasing its susceptibility to hydrolysis.

In general, differences in digestibility were seen, probably related to variations in the amylose/amylopectin ratio, granular size and organization, degree of crystallinity, and possible interactions with other matrix components.

Therefore, studying the rate and extent of starch hydrolysis commonly referred to as hydrolysis kinetics can contribute to the identification of genotypes or the adjustment of processing parameters to achieve specific glucose release rates (Dhital et al., 2016). The results show that the cooking conditions used in this study were relatively harsh, causing partial or total degradation of resistant starch. Therefore, it is recommended to evaluate alternative cooking times and temperatures that allow for the preservation of higher levels of resistant starch.

### 3.4 Content of organic acids and soluble sugars

The marked increase in soluble sugars between the unripe and ripe stages confirms the climacteric nature of the fruit. Total sugar content (raffinose + sucrose + glucose + fructose) increased in all cultivars, reaching maximum values in the raw, ripe fruit, particularly in Guineo (761.68 mg/g db), followed by Guayabo (570.51 mg/g db), Dominico Harton (563.52 mg/g db), and Cachaco (482.51 mg/g db) (Table 3). This behavior is consistent with the postharvest degradation of reserve starch, the main component of dry matter in unripe plantains, which is progressively hydrolyzed by the coordinated action of amylolytic enzymes.

During ripening, sucrose acts as a transient metabolic intermediate; However, glucose and fructose predominated in ripe fruit, while sucrose represented a minor fraction. In raw ripe Dominico Harton, for example, glucose (270.01 mg/g db) and fructose (280.90 mg/g db) far exceeded sucrose (7.93 mg/g db). This pattern suggests high activity of invertases responsible for the hydrolysis of sucrose into hexoses, with acid invertase being one of the main regulators of sucrose levels during banana ripening (Fils-Lycaon et al., 2011). Ripening also significantly affected the concentration of organic acids (oxalic, citric, malic, and succinic, Table 3), with increases dependent on the genotype. In Guayabo, for example, malic acid increased from 7.13 to 29.36 mg/g db and citric acid from 14.64 to 29.63 mg/g db. Similar results have been reported in other banana genotypes, where malate and citrate accumulation is seen at mature stages (Gao et al., 2025). This behavior is associated with the intensification of mitochondrial respiration during the climacteric peak. Since these compounds are intermediates of the tricarboxylic acid cycle, their accumulation reflects a high respiratory flux and active carbon redistribution. Malate can function as a carbon reservoir and modulator of cellular redox balance. Taking together, the simultaneous increase in sugars and organic acids contributes to the characteristic sensory profile of ripe bananas (Al-Dairi et al., 2023).

Thermal processing induced significant modifications in the carbohydrate profile. In all cultivars, cooking the unripe fruit increased sucrose concentration and reduced glucose and fructose concentrations. This behavior can be explained by starch gelatinization, the onset of which, according to the data from the evaluated flours (Table 4), ranges between 61 and 69.4 °C. This process involves the loss of the granule's crystalline structure, which favors the release and greater extractability of carbohydrates previously kept in the cell matrix. Additionally, the thermal inactivation of invertases limits the hydrolysis of sucrose to hexoses, promoting their relative accumulation. In contrast, monosaccharides, due to their high-water solubility, tend to leach during boiling, which explains their decrease after cooking, a consistent behavior in both unripe and ripe samples. About organic acids, cooking caused significant reductions in malic, succinic, and citric acids, suggesting migration into the aqueous medium. Oxalic acid, on the other hand, showed relative increases, possibly associated with concentration effects due to the loss of other soluble solids or its release from cellular compartments.

From a nutritional standpoint, changes in the total sugar profile have direct implications for the glycemic index (eGI) of the fruit. Therefore, flours obtained from fresh, ripe fruit, characterized by a higher concentration of free hexoses, have a higher glycemic index than those from unripe fruit (Table 2). Furthermore, boiling, by reducing the concentration of monosaccharides through leaching and changing the starch matrix, could alter the availability of digestible carbohydrates and, so, the glycemic response.

### 3.5 Thermal properties of gelatinization measured in flour

In this study, the Dominico Harton exhibited the highest amylose content in the unripe state (18.7%), which coincided with relatively high gelatinization enthalpy (ΔH) value. This behavior suggests a higher proportion of crystalline domains or a more compact amylopectin organization in these materials. In the raw flour, ripening caused a consistent increase in the onset (To) gelatinization temperatures in the Dominico Harton, Cachaco, and Guayabo cultivars (Table 4). This increase suggests greater thermal stability of residual starch in the mature state, possibly associated with a structural reorganization of the granules during progressive starch degradation. Gelatinization temperatures are closely related to the stability of the starch's crystalline regions, formed mainly by amylopectin double helices; therefore, higher to values indicate greater structural stability of the granular system (Hoover, 2001). Furthermore, the reduction in the total starch fraction and changes in its molecular organization during ripening contribute to the observed modifications in the thermal properties of residual starch (Cordenunsi-Lysenko et al., 2019). The most notable effect of ripening was the significant reduction in the enthalpy of gelatinization (ΔH). In most of the cultivars evaluated, ΔH decreased between 59 and 65% when transitioning from the unripe to the ripening state, while no endothermic transitions were detected in Guineo. Since enthalpy reflects the energy required to melt the crystalline regions of amylopectin, this reduction indicates a substantial loss of structural order and/or a decrease in native starch content (Chakraborty et al., 2022; Cooke & Gidley, 1992). These changes are associated with starch degradation and decreased granule crystallinity during fruit ripening, a process that leads to the conversion of starch into soluble sugars.

No detectable endothermic transitions were recorded in the boiled samples, confirming the loss of crystalline structure after heat treatment. The absence of peaks in the DSC analysis indicates that the gelatinization process was completed during boiling, resulting in irreversible disruption of the starch's molecular order. Finally, the thermal response of starch depends simultaneously on the genotype, the physiological state of the fruit, and the treatment applied. This indicates that structural changes induced by ripening are not uniform across cultivars and that their susceptibility to thermal modification varies differentially.

### 3.6 Pasting and functional properties

The cultivar significantly affected all filling parameters (*p* < 0.05); The highest *p* value was observed in the breakdown (*p* = 0.0117), while in the other parameters it was *p* ≤ 0.001. Processing also had a significant effect (*p* < 0.01), with *p* = 0.001 for pasting temperature, maximum viscosity, minimum viscosity, breakdown and setback, and *p* < 0.001 for final viscosity. The effect of the maturity state could only be statistically evaluated on the final viscosity (*p* < 0.001), since no viscosity peak was detected in the mature samples; For the other pasting parameters, their effect is described as an observation (absence of viscosity peak) and not as a statistical test. Viscosity profiles changed markedly during ripening and cooking (Fig. 2), reflecting structural modifications in starch composition and functionality.

**Fig. 2:**
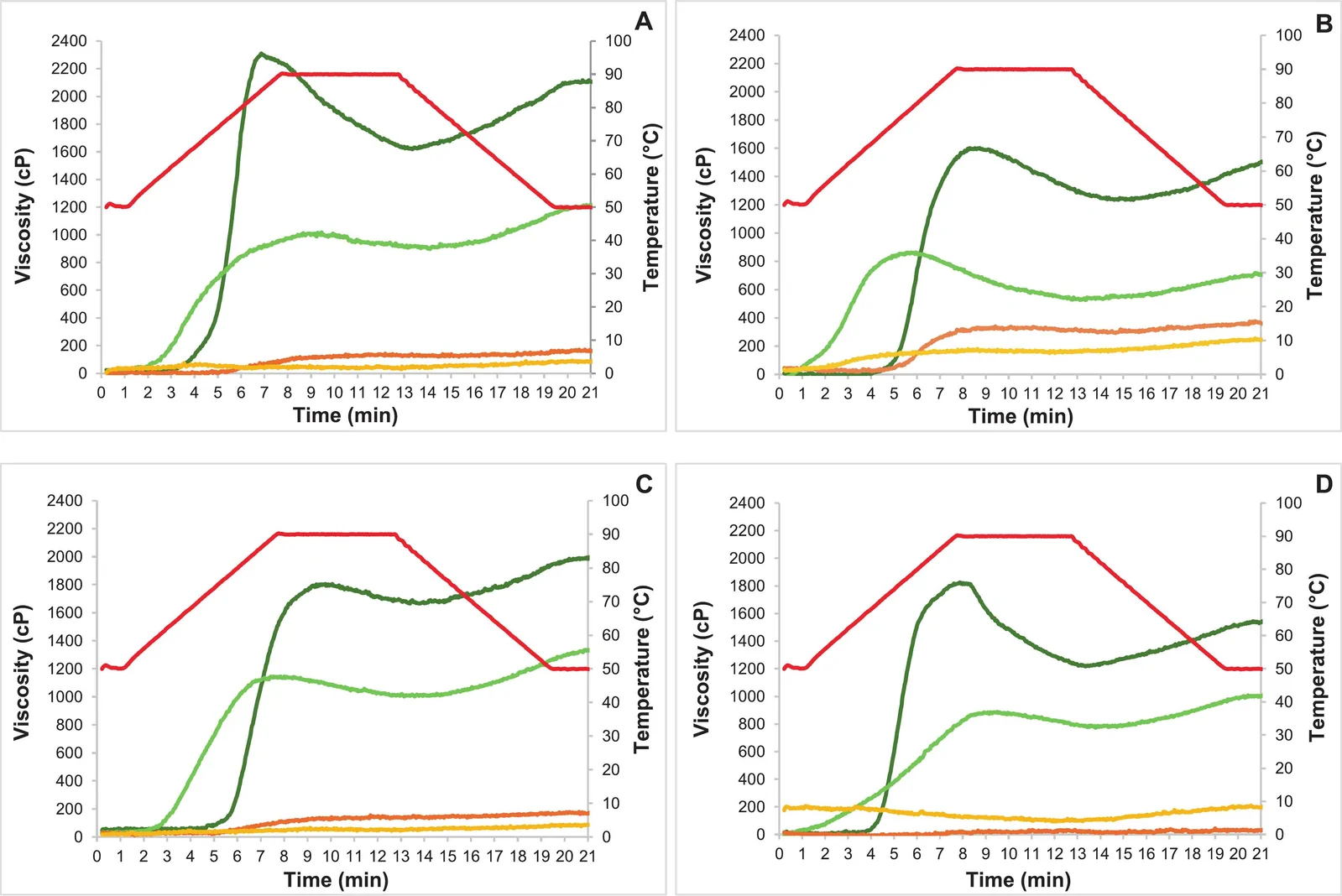
Pasting curve of raw and boiled, cooking banana flour at different ripening stages using rapid viscoanalyser. A: Dominico Harton; B: Cachaco; C: Guayabo; D: Guineo; **−--** Unripe+Raw; **−--** Unripe+ Boiled; **−--** Ripe+Raw; **−--** Ripe+Boiled.

Peak viscosity ranged from 875 to 2297 cP, with the highest value observed in Dominico Harton (Unripe+Raw), these results suggest that flours with higher starch levels develop stronger paste structures and may be helpful in products requiring thick textures. Indeed, the overall trend in peak viscosity was consistent with starch content across samples.

In contrast, Ripe+Raw and Ripe+Boiled samples did not show defined peak or minimum viscosity during heating, preventing calculation of breakdown and setback values. Ripe+Boiled samples displayed a nearly linear RVA profile. At the evaluated concentration (8%), starch granules were highly dispersed, limiting the development of a viscosity peak. Additionally, increased sugar levels resulting from starch hydrolysis likely restricted starch swelling, as water preferentially interacts with soluble sugars rather than starch polymers (Glicksman, 1969).

Pasting temperature ranged from 54.4 °C to 78.9 °C. Guayabo (Unripe+Raw) showed the highest temperature (78.9 °C), suggesting greater thermal resistance, while Guineo (Unripe+Boiled) showed the lowest value (54.4 °C), indicating easier gelatinization. In general, pasting temperature decreased with ripening and cooking, except in Cachaco, which showed a slight increase during ripening. This behavior may be attributed to higher sugar concentrations and other low-molecular-weight compounds competing with starch for water (Ayo-Omogie et al., 2021). In cooked samples, estimation of pasting temperature was more difficult due to slight initial increases in viscosity associated with baseline instability.

Final viscosity decreased with ripening and processing and differed significantly among cultivars (*p* < 0.0001). Dominico Harton (Unripe+Raw) presented with the highest final viscosity, while Guineo (Ripe+Raw) showed the lowest. These differences are likely related to amylose content, as higher amylose concentrations promote stronger gel formation, consistent with previous reports (Wani et al., 2010). Flours with higher final viscosity may therefore be suitable for bakery applications requiring stable and viscous dough systems.

Breakdown viscosity, which reflects paste stability under heating and shear, ranged from 102 to 696 cP. Unripe+Raw flours exhibited higher breakdown values, indicating greater susceptibility to structural disintegration, while Unripe+Boiled flours showed lower values, suggesting improved stability under high-temperature processing. Setback viscosity, an indicator of retrogradation tendency, was also higher in Unripe+Raw samples, implying greater susceptibility to retrogradation and syneresis. Since retrogradation is influenced by amylose and amylopectin content, molecular structure, temperature, and pH, differences among cultivars are expected. Among Unripe+Raw samples, Cachaco (13.0% amylose) exhibited a lower setback value (265 cP) than Dominico Harton (17.3% amylose; 482 cP), indicating reduced retrogradation and improved technological stability, likely due to its lower amylose content (Felisberto et al., 2020). Lower setback values are particularly desirable in products requiring reduced syneresis, such as frozen or baked goods. Overall, Unripe+Raw and Unripe+Boiled flours produced firmer pastes with greater retrogradation tendency, while Ripe+Raw and Ripe+Boiled flours showed more stable behavior during mixing, heating, and cooling, mainly due to their lower starch content (Bakare et al., 2017).

Solubility and swelling power were significantly affected (*p* < 0.001) by cultivar (C), ripening stage (R), processing (P), and their three-way interaction (C × R × P). These results indicate that flour functionality does not depend on a single factor, but rather on the interaction between genotype, ripeness stage, and heat treatment. Ripening resulted in a marked decrease in amylose content in all cultivars, with a particularly pronounced effect in Guineo (from 14.8% to 2.3% in the raw state). This reduction was associated with a significant increase in solubility in the untreated ripe samples (up to 76.5%), a phenomenon attributable to starch hydrolysis and the consequent accumulation of soluble sugars. In contrast, the Unripe+Raw samples, characterized by higher amylose content (13–18%), showed low solubility values (11–15%), suggesting a more organized granular structure with a greater degree of intermolecular association and lower availability of soluble fractions. Thermal processing significantly modified these relationships. In the unripe state, boiling increased the amylose content, as well as solubility and swelling power in most cultivars (for example, in Cachaco: from 8.9 to 16.9 g/g). This behavior suggests the occurrence of partial gelatinization and greater exposure of hydrophilic groups, favoring interaction with water. In cooked ripe samples, the effect was cultivar-dependent: while solubility increased after boiling in Cachaco and Guineo, it decreased in Dominico Harton and Guayabo compared to the raw ripe samples. This differential response could be explained by leaching processes of soluble components during cooking and by molecular reorganization phenomena following heat treatment.

Overall, amylose content showed an inverse relationship with solubility, although heat processing is a determining factor in the expression of these properties. The results confirm that ripening promotes starch solubilization, while boiling induces physicochemical transformations dependent on both the stage of ripeness and the cultivar, conditioning the functional behavior of the flours and their potential technological applications.

### 3.7 Optical, polarized and scanning electron (SEM) microscopy of flour

Combined analysis using optical microscopy, polarized light microscopy, and scanning electron microscopy (SEM) enabled a comprehensive assessment of starch structural changes in banana flours as influenced by cultivar, ripening stage, and heat treatment (Fig. 3, Fig. 4).

**Fig. 3:**
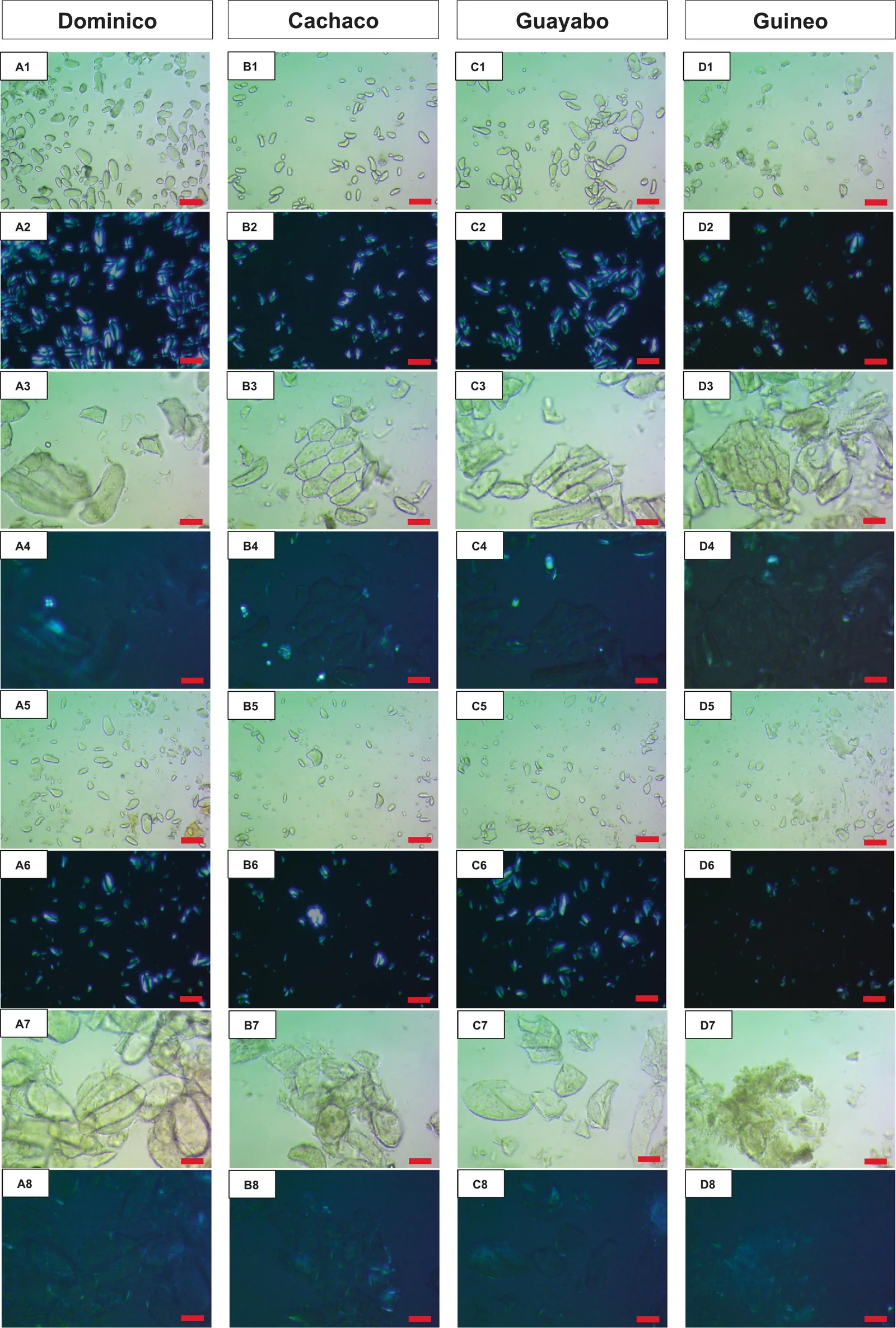
Normal light, polarized light (magnitude 20×) microscopy of Dominico Harton (A), Cachaco (B), Guayabo (C) and Guineo (D) flours. A1-D1: Normal light of Unripe+Raw; A2-D2: polarized light of Unripe+Raw; A3-D3: normal light of Unripe+Boiled; A4-D4: polarized light of Unripe+Boiled; A5-D5: normal light of Ripe+Raw; A6-D6: polarized light of Ripe+Raw; A7-D7: normal light of Ripe+Boiled; A8-D8: polarized light of Ripe+Boiled. Scale bar: 20 μm.

**Fig. 4:**
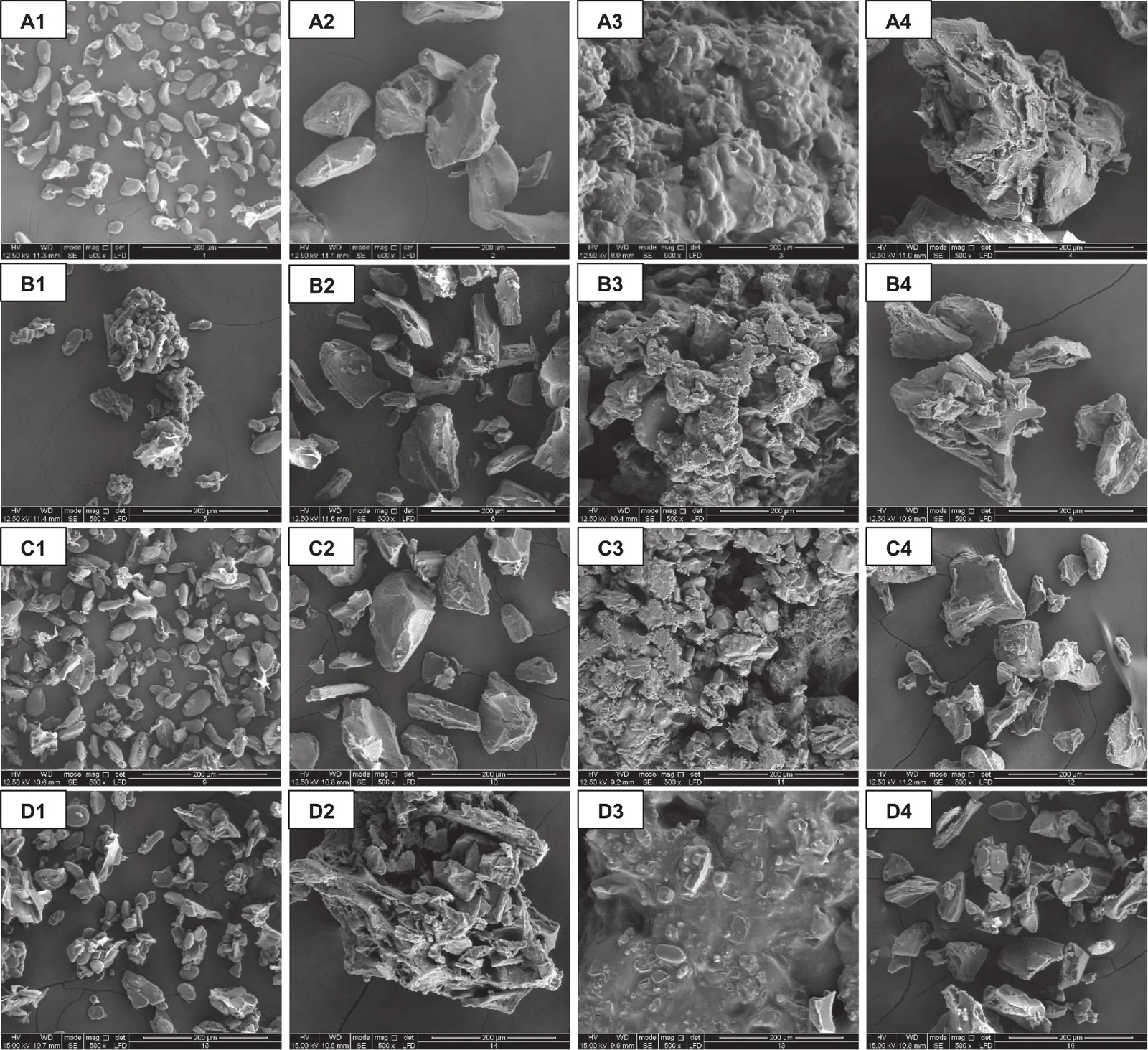
Scanning electron (SEM-magnitude 200×) microscopy of Dominico Harton (A), Cachaco (B), Guayabo (C) and Guineo (D) flours. A1-D1: Unripe+Raw; A2-D2: Unripe+Boiled; A3-D3: Ripe+Raw; A4-D4: Ripe+Boiled.

In unripe and raw samples, SEM micrographs (A1, B1, C1, and D1, Fig. 4) showed discrete starch granules with oval to ellipsoidal morphology and smooth surfaces. This was consistent with optical microscopy observations and with the strong birefringence detected under polarized light (A2, B2, C2, and D2, Fig. 3), evidenced by the Maltese cross pattern, indicative of the semicrystalline organization of native starch (Zhang et al., 2005). This structural integrity aligns with the high total starch content (67.5–88.8%, db) and the predominance of resistant starch (RS), particularly in Dominico Harton. The presence of intact crystalline granules restricts enzymatic accessibility, explaining the low digestibility of native starch in unripe plantains (Ovando-Martínez et al., 2009).

Thermal treatment induced extensive structural disruption. SEM images (A2, B2, C2, and D2, Fig. 4) revealed compact aggregates and amorphous matrices resulting from granule swelling and collapse, consistent with optical microscopy and the loss of birefringence observed under polarized light (A3–A4, Fig. 3). This transition from ordered to amorphous structure constitutes direct evidence of starch gelatinization. Consequently, digestibility shifted markedly, with a substantial increase in rapidly digestible starch (RDS) and a drastic reduction in RS, confirming that gelatinization enhances enzymatic susceptibility.

Ripening further modified starch structure through enzymatic degradation. In raw samples, SEM (A3, B3, C3, and D3, Fig. 4) and optical images (A5, B5, C5, and D5, Fig. 3) showed reduced granule density and a more fragmented matrix, particularly in Guineo, reflecting starch hydrolysis. Despite this degradation, residual birefringence (A6, B6, C6, and D6, Fig. 3) indicates partial preservation of crystalline domains. This explains the concurrent decrease in total starch and resistant starch fractions during maturation.

The combined effect of ripening and cooking produced the most pronounced structural transformation. SEM images (A4, B4, C4, and D4, Fig. 4) showed fully collapsed, irregular aggregates, while polarized light microscopy (A8, B8, C8, and D8, Fig. 3) confirmed the complete loss of birefringence. Under these conditions, RS approached negligible levels and RDS became dominant. Thus, starch digestibility is directly governed by microstructural organization: intact crystalline granules favor RS formation, while gelatinization and enzymatic breakdown promote rapidly digestible fractions.

### 3.8 Multivariate analysis of the physicochemical and quality characteristics of the evaluated genotypes

Pearson correlation analysis revealed strong integration among the compositional, structural, and functional domains of the system, confirming the multivariate patterns observed in the principal component analysis (PCA).

A total of 39 parameters were evaluated, obtained through experimental quantification and predictive estimations, allowing for the exploration of relationships between carotenoids, starch structure, digestibility, pasting properties and thermal behavior, consequently, PCA was performed on the eleven key physicochemical and nutritional variables (t-bC, TS, RS, Amy, eGI, Sol, SP, Malic, Suc, Sug, and To). The correlation matrix (Fig. 5A) revealed associations consistent with the structural gradients identified in PCA (Fig. 5B). Provitamin A carotenoids showed almost perfect collinearity, particularly between trans-β-carotene and RAE (*r* = 0.99) and between trans-β-carotene and pVACs (*r* = 0.96), confirming that this compound is the main determinant of provitamin A potential in the evaluated *Musaceae*.

**Fig. 5:**
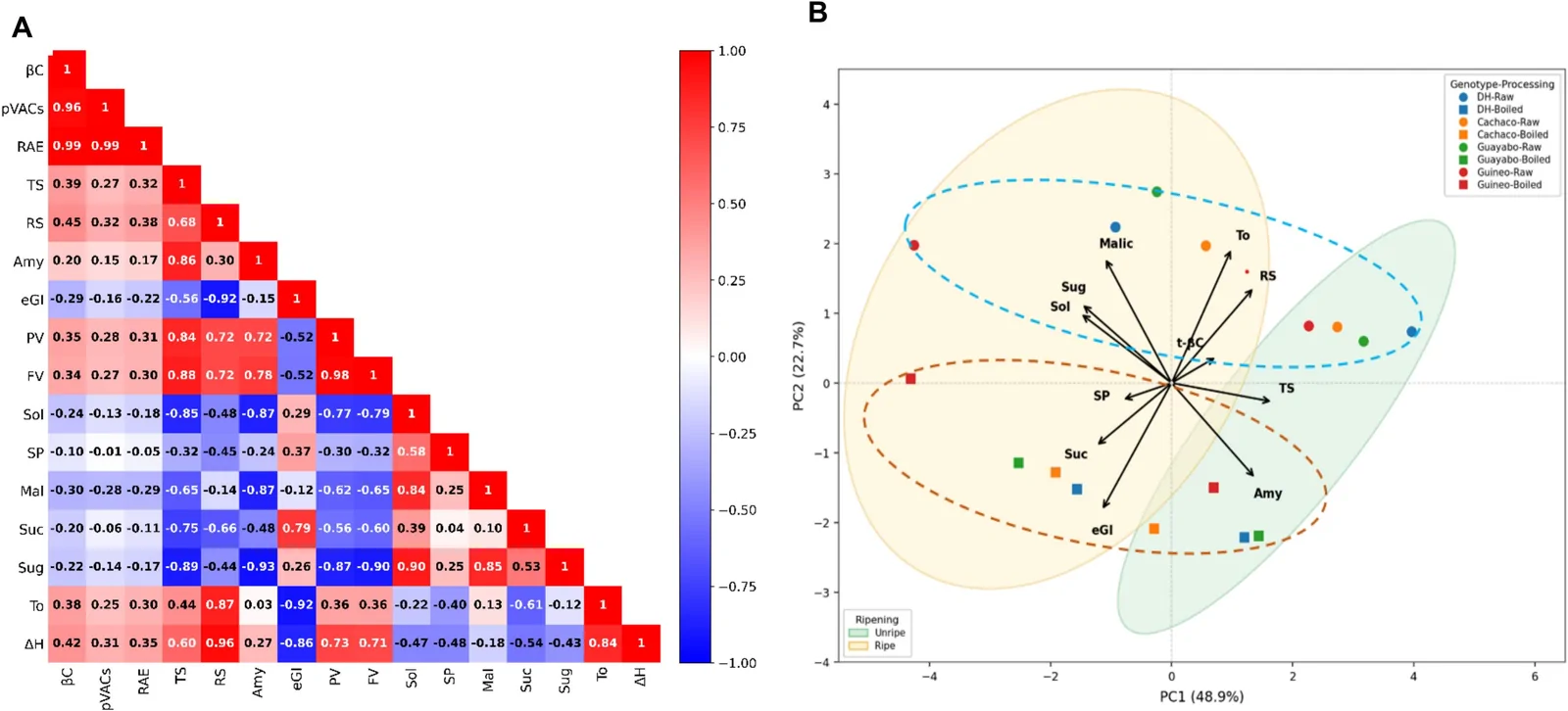
Multivariate analysis of physicochemical variables. A: Pearson correlation matrix and B: principal component analysis (PCA) biplot. βC: all-trans beta-carotene; pVACs: provitamin A carotenoids; RAE: retinol activity equivalent; TS: total starch; RS: resistant starch; Amy: amylose; eGI: estimated glycemic index; PV: peak viscosity, FV: final viscosity; Sol: Solubility; SP: Swelling power, Mal: malic acid; Suc: sucrose; Sug: total sugar; To: onset temperature; ∆H: gelatinization enthalpy. ●: Raw; ■: Boiled; __: Unripe; __: Ripe; _ _ _: Raw; _ _ _: Boiled.

Compositional transformations were dominated by the conversion of starch into soluble sugars, shown by the strong negative correlations between total starch and total sugars (*r* = −0.89) and between amylose and total sugars (*r* = −0.93). This structural process was also reflected in the functional behavior of starch: peak viscosity (PV) was negatively correlated with total sugars (PV vs. Sug, *r* = −0.87), showing that starch hydrolysis reduces granular swelling and limits viscosity development during gelatinization. Consistently, sucrose showed a positive correlation with the estimated glycemic index (Suc vs. eGI, *r* = 0.79), reflecting the effect of simple sugars on glycemic potential. Resistant starch (RS) appeared as a key structural determinant of digestibility and thermal stability. This component showed a strong negative correlation with the estimated glycemic index (RS vs. eGI, *r* = −0.92) and positive correlations with the enthalpy of gelatinization (ΔH) obtained by DSC (*r* = 0.96), suggesting that a higher RS content is associated with more organized crystalline domains that require more energy to gelatinize and are less susceptible to enzymatic hydrolysis. This behavior is consistent with the previously described relationship between starch crystallinity, thermal stability, and digestibility (Tester et al., 2004).

Principal component analysis (PCA) based on key physicochemical variables showed that the first two components explained 71.6% of the total variance (Fig. 5B). The distribution of scores clearly separated the samples according to maturation stage (ripe vs unripe, PC1) and processing (raw vs. boiled, PC2), indicating that these factors are determinants of variability. PC1, which accounted for the largest proportion of variance, represented a starch transformation axis, characterized by the degradation of structural polysaccharides and the concurrent accumulation of soluble sugars. This was supported by strong negative correlations between total starch, amylose, and total sugars, as well as their association with viscoamylographic properties and the estimated glycemic index. In contrast, PC2 captured secondary variation related to the nutritional and structural quality of the matrix, particularly driven by provitamin A carotenoids and resistant starch organization. These variables were also positively associated with thermal stability, indicating that samples with higher structural integrity retain both crystalline order and nutritional compounds. Taken together, these results show that the variability observed among genotypes, stages and maturation and processing conditions is mediated by dominant structural processes such as progressive starch hydrolysis and accumulation of soluble sugars, structural reorganization of starch and changes in carotenoid content, which simultaneously modulate the nutritional, digestive, thermal and functional properties of the system.

### 3.9 Methodological considerations and scope

Despite the comprehensive physicochemical characterization presented, several limitations must be acknowledged. First, starch and functional analyses (Table 2, Table 3, Table 4) were conducted in analytical duplicate (*n* = 2) derived from a single pooled pulp per treatment combination rather than from three independent biological bunches, limiting the statistical power of multiple comparisons for those variables. Second, all four cultivars were harvested from a single experimental site during a single season (San Isidro, Palmira, Valle del Cauca, Colombia), which restricts the generalisability of the results to other edaphoclimatic conditions; multi-environment evaluation would be needed to partition genotype-by-environment interactions. Third, carotenoid bioaccessibility was not directly measured; all carotenoid quantification was performed by solvent extraction and HPLC-DAD, and inferences about bioaccessibility are based on literature precedent rather than experimental evidence. Fourth, only boiling in water was evaluated as a thermal treatment; steaming, frying, and other culturally relevant cooking methods may differentially affect carotenoid stability, starch digestibility, and functional properties, and their evaluation is recommended in future work.

## 4 Conclusion

This study demonstrates that genotype, ripening stage, and thermal processing significantly influence the nutritional and functional properties of cooking bananas cultivated in Colombia. Dominico Harton showed the highest provitamin A carotenoid content, highlighting its potential nutritional relevance in regions affected by vitamin A deficiency. Unripe flours were characterized by high resistant starch content and low estimated glycemic index, confirming their potential as functional ingredients with beneficial metabolic effects. In contrast, ripening and boiling significantly increased starch digestibility and glycemic potential due to starch gelatinization and enzymatic hydrolysis. Ripening also promoted a marked accumulation of soluble sugars and organic acids, contributing to the characteristic sensory and metabolic profile of ripe bananas.

These findings highlight the potential of unripe cooking banana flour, particularly from cultivars rich in resistant starch and provitamin A carotenoids, as a functional food ingredient with beneficial metabolic properties, while acknowledging that contributions to dietary vitamin A status and food security at the population level require validation through bioavailability, consumption, and dietary intervention studies.

## CRediT authorship contribution statement

**Jhon Larry Moreno-Alzate:** Writing – original draft, Methodology, Investigation, Data curation, Conceptualization. **Santiago Arufe:** Writing – review & editing, Investigation, Formal analysis, Data curation. **Thierry Tran:** Writing – review & editing, Project administration, Methodology, Investigation. **Moralba Dominguez:** Validation, Methodology, Investigation, Data curation. **Dominique Dufour:** Writing – review & editing, Validation, Supervision, Project administration, Methodology, Investigation, Funding acquisition, Formal analysis, Conceptualization.

## Declaration of competing interest

The authors declare that they have no known competing financial interests or personal relationships that could have appeared to influence the work reported in this paper.

## Data Availability

Data will be made available on request.
