# Supplementary material for: Ripening and thermal processing modulate starch digestibility, techno-functional properties and carotenoid profile in cooking banana cultivars (Musa spp.)
Source: Food Chem X. 2026 Oct 27;40:104678. doi: 10.1016/j.fochx.2026.104678 (PMC13760482; doi:10.1016/j.fochx.2026.104678)
Supplement: Supplementary file 1 [file mmc1.docx]

**Ripening and thermal processing modulate starch digestibility, techno-functional properties and carotenoid profile in cooking banana cultivars (*Musa* spp.)**

**Jhon Larry Moreno-Alzate**^1^**, Santiago Arufe**^2, 3^**, Thierry Tran**^1, 2, 3^**, Moralba Dominguez**^1^**, Dominique Dufour** ^2, 3^

**^1^** The Alliance of Bioversity International and the International Center for Tropical Agriculture (CIAT), Apartado Aereo, Cali 6713, Colombia

**^2^** CIRAD, UMR Qualisud, F-34398 Montpellier, France.

**^3^** Qualisud, Avignon Université, CIRAD, Institut Agro, IRD, Université de Montpellier, Université La Réunion, Montpellier, France.

**Corresponding author**: j.l.moreno@cgiar.org, [dominique.dufour@cirad.fr](mailto:dominique.dufour@cirad.fr)

a

| Resistant starch content (%, db) | Amylose content (%, db) |
| --- | --- |
| 70.9168 | 18.6827 |
| 71.2715 | 18.6827 |
| 29.5902 | 7.02618 |
| 28.776 | 6.46048 |
| 53.0452 | 16.6184 |
| 51.4972 | 16.6184 |
| 33.284 | 8.64718 |
| 33.6958 | 9.07161 |
| 63.1544 | 15.4628 |
| 63.3633 | 15.4628 |
| 36.5093 | 5.91154 |
| 38.7166 | 7.51993 |
| 52.3715 | 11.8876 |
| 52.3612 | 11.8876 |
| 6.26806 | 2.05895 |
| 4.96425 | 2.01258 |

b

| Resistant starch content (%, db) | Amylose content (%, db) |
| --- | --- |
| 70.9168 | 18.6827 |
| 71.2715 | 18.6827 |
| 1.17939 | 18.3477 |
| 3.07319 | 19.092 |
| 29.5902 | 7.02618 |
| 28.776 | 6.46048 |
| 0.509895 | 11.4274 |
| 0.54211 | 11.734 |
| 53.0452 | 16.6184 |
| 51.4972 | 16.6184 |
| 3.15489 | 15.4285 |
| 1.63632 | 14.6568 |
| 33.284 | 8.64718 |
| 33.6958 | 9.07161 |
| 0.368556 | 9.99209 |
| 0.401498 | 9.73518 |
| 63.1544 | 15.4628 |
| 63.3633 | 15.4628 |
| 13.1583 | 19.6414 |
| 15.0654 | 18.5107 |
| 36.5093 | 5.91154 |
| 38.7166 | 7.51993 |
| 0.288397 | 6.71021 |
| 0.301308 | 6.24659 |
| 52.3715 | 11.8876 |
| 52.3612 | 11.8876 |
| 19.9159 | 13.7674 |
| 17.8054 | 14.5431 |
| 6.26806 | 2.05895 |
| 4.96425 | 2.01258 |
| 0.433158 | 3.06031 |
| 0.434554 | 3.13047 |

**Figure S1 (supplementary figure)**. Correlation analysis between amylose and resistant starch content. A). Correlation between amylose and resistant starch content in flours obtained from unripe and ripe samples. B). Correlation between amylose and resistant starch content in flours obtained from green and mature samples, both raw and boiled.
